# Structure–Activity Relationship of Synthetic Ginkgolic Acid Analogs for Treating Type 2 Diabetes by PTPN9 Inhibition

**DOI:** 10.3390/ijms23073927

**Published:** 2022-04-01

**Authors:** Jinsoo Kim, Jinyoung Son, Dohee Ahn, Gibeom Nam, Xiaodi Zhao, Hyuna Park, Woojoo Jeong, Sang J. Chung

**Affiliations:** 1 School of Pharmacy, Sungkyunkwan University, Suwon 16419, Korea; neto543@naver.com (J.K.); ehgml94@naver.com (D.A.); skarlqja12@skku.edu (G.N.); zhaoxiaodi1019@gmail.com (X.Z.); 2 Department of Biopharmaceutical Convergence, Sungkyunkwan University, Suwon 16419, Korea; qoxmfspt753@naver.com (J.S.); hyunaaa_1226@naver.com (H.P.); jwjoo5@naver.com (W.J.)

**Keywords:** ginkgolic acid analogs, PTPN9 inhibitors, glucose uptake, insulin sensitivity, AMPK, Akt

## Abstract

Ginkgolic acid (C13:0) (GA), isolated from Ginkgo biloba, is a potential therapeutic agent for type 2 diabetes. A series of GA analogs were designed and synthesized for the evaluation of their structure–activity relationship with respect to their antidiabetic effects. Unlike GA, the synthetic analog **1e** exhibited improved inhibitory activity against PTPN9 and significantly stimulated glucose uptake via AMPK phosphorylation in differentiated 3T3-L1 adipocytes and C2C12 myotubes; it also induced insulin-dependent AKT activation in C2C12 myotubes in a concentration-dependent manner. Docking simulation results showed that **1e** had a better binding affinity through a unique hydrophobic interaction with a PTPN9 hydrophobic groove. Moreover, **1e** ameliorated palmitate-induced insulin resistance in C2C12 cells. This study showed that **1e** increases glucose uptake and suppresses palmitate-induced insulin resistance in C2C12 myotubes via PTPN9 inhibition; thus, it is a promising therapeutic candidate for treating type 2 diabetes.

## 1. Introduction

Type 2 diabetes mellitus (T2DM) is a progressive metabolic disorder characterized by hyperglycemia resulting from the combination of resistance to insulin action, inadequate insulin secretion, and excessive or inappropriate glucagon secretion [1,2,3]. Long-term hyperglycemia is an important cause of mortality and morbidity worldwide. The WHO assumes that diabetes will be the seventh leading cause of fatality by 2030 [4]. Strict glycemic control for T2DM patients is, therefore, of paramount importance.

Protein tyrosine phosphatases (PTPs) constitute a large family of signaling enzymes that are involved in numerous cellular processes, such as growth, differentiation, metabolism, and immune response [5]. More specifically, PTPs such as PTPN1, PTPN2, PTPN9, PTPN11, PTPRF, and DUSP9 are associated with the pathogenesis of type 2 diabetes, as they negatively modulate insulin signaling and induce insulin resistance [6,7,8,9,10,11,12].

*Ginkgo biloba* (Ginkgo), also known as the maidenhair tree, is a deciduous tree, which has existed on Earth for approximately 200 million years and is considered to be a “living fossil” [13]. Its extracts constitute some of the most widely used herbal medicines and dietary supplements worldwide, where the sales of the related products have been growing at a rapid rate of 25% per year in the commercial market [14]. The findings of several studies have shown that ginkgo extracts possess significant pharmacological and clinical effects, such as free radical scavenging effects [15], blood flow-improving effects [16], protective effects against myocardial ischemia [17,18], cognitive enhancement effects [13,19], and tumor cell apoptosis-promoting effects [20]. In a previous study, we found that ginkgolic acid (C13:0) (GA) inhibits PTPN9 and DUSP9, thereby stimulating AMPK phosphorylation and increasing glucose uptake [21]. In addition, the anticancer and tyrosinase inhibitory effects of GA analogs have been evaluated, and they have been found to exhibit significant biological activities [22,23,24]. Hence, GA analogs are considered to be interesting compounds for drug discovery studies.

In a previous study, we verified whether PTPN9 and DUSP9 knockdown induce an upregulation in the phosphorylation of AMPK [21], a crucial marker involved in the translocation of glucose transporter 4 (GLUT4) to the plasma membrane. PTPN9 knockdown induced a greater increase in AMPK phosphorylation than DUSP9 knockdown. Concurrent PTPN9 and DUSP9 knockdown yielded additive effects on AMPK phosphorylation. Furthermore, PTPN9 inhibition also induces insulin sensitization in diet-induced obese mice [25]. However, since DUSP9 plays a negative role in induced insulin resistance [26], the significant inhibition of DUSP9 in the long term may induce insulin resistance in target cells. Therefore, we hypothesized that compounds with significant PTPN9 inhibitory effects and moderate or no DUSP9 inhibitory effects would act as good antidiabetic drug candidates.

In this study, we aimed to design synthetic GA analogs that can be easily synthesized and discover antidiabetic compounds with an improved biological activity through a structure–activity relationship study. Hence, GA-resembling alkyloxy salicylic acids (ASAs) were designed (benzylic CH_2_ groups in GAs are replaced with ether oxygen atoms), synthesized via Williamson ether synthesis, and tested for their inhibitory effects against PTPN9 and DUSP9. Subsequently, the antidiabetic effects of the selected compounds were evaluated using a cell-based assay to demonstrate the usefulness of the design rationale that might yield a good drug candidate.

## 2. Results

### 2.1. Synthesis of the Designed Compounds

**Figure 1:**
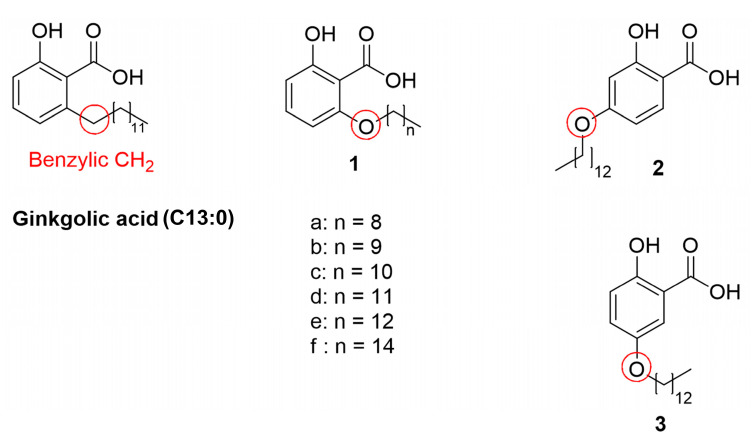
Structures of GA and alkyloxy salicylic acids.

**Scheme 1:**
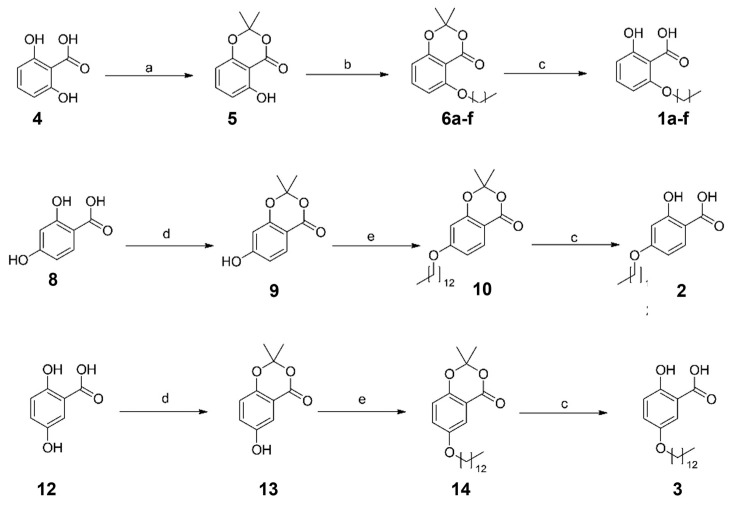
Synthesis of the designed compounds. Reagents and conditions: (a) acetone, SOCl_2_, DMAP, DME; (b) 1-bromoalkane, K_2_CO_3_, DMF, 60 °C; (c) KOH, THF, 60 °C; (d) acetone, TFAA, TFA; (e) 1-bromotridecane, K_2_CO_3_ or Cs_2_CO_3_, DMF, 60 °C.

In previous studies, GA derivatives were synthesized using Suzuki coupling and the resulting alkene reduction as the key steps [23,24]. Provided that the alkyl chains in GAs enhanced their binding affinity to the enzymes through hydrophobic interactions, the benzylic CH_2_ group was replaced with O in the target design (Figure 1). This facilitated the synthesis of the designed compounds (**1**–**3**) derived from dihydroxy benzoic acids (**4**,**8**,**12**), as shown in Scheme 1. The carboxyl and 2-hydroxyl groups of the dihydroxybenzoic acids were selectively protected by acetonide formation followed by the alkylation of the remaining hydroxyl group via Williamson ether synthesis and acetonide deprotection. More specifically, 2,6-dihydroxybenzoic acid (**4**) was protected to form **5** through acetonide formation in the presence of thionyl chloride and 1,2-dimethoxyethane (DME). With 2,4-Dihydroxybenzoic acid (**8**) and 2,5-dihydroxybenzoic acid (**12**), acetonide formation was not possible under the same reaction conditions. However, the desired protection of **8** and **12** was achieved through treatment with trifluoroacetic anhydride and acetone in trifluoroacetic acid. The remaining free hydroxyl (OH) groups were reacted with appropriate alkyl halides to form **3**, **7**, or **11,** which were deprotected using KOH to obtain the designed products (**1**–**3**) in quantitative yields. Details of the synthesis process and spectral data for each compound are presented in the Supplementary Materials Section.

### 2.2. Structure–Activity Relation of the Synthesized GA Analogs with Respect to PTPN9 and DUSP9 Inhibition

**Figure 2:**
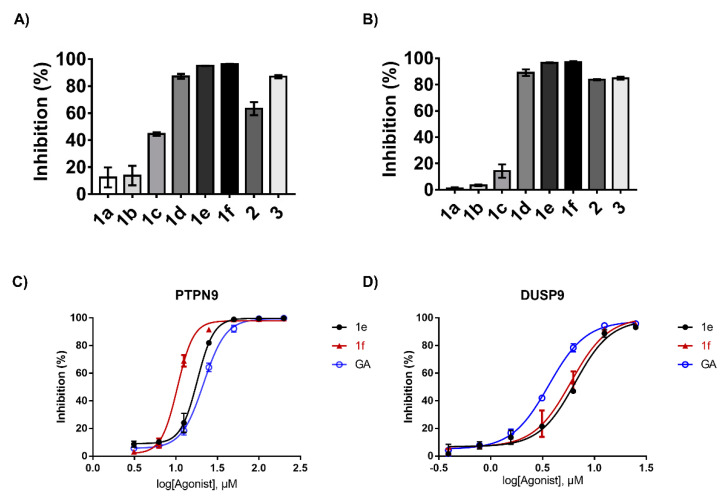
Inhibitory activities of GA analogs against PTPN9 (**A**) and DUSP9 (**B**). For each compound, the final enzyme concentration was 50 µM and 20 µM for PTPN9 and DUSP9, respectively. PTPN9 (0.15 nM) and DUSP9 (200 nM) were added to the mixture consisting of DiFMUP (220 µM for PTPN9 and 90 µM for DUSP9) and each compound (50 µM for PTPN9 and 20 µM for DUSP9). Enzyme activity was measured by monitoring the change in fluorescence intensity at Ex/Em = 365/450 nm. Enzyme inhibitory effects were evaluated by comparing enzyme activity in the presence of DMSO as a control, as well as each compound dissolved in DMSO. Determination of IC_50_ values for **1e, 1f**, and GA against PTPN9 (**C**) and DUSP9 (**D**). The inhibitor concentrations used to determine IC_50_ were 200, 100, 50, 25, 12.5, 6.25, or 3.125 µM for PTPN9 and 25, 12.5, 6.25, 3.125, 1.563, 0.782, or 0.391 µM for DUSP9. Data are presented as mean ± standard deviation (*n* = 2).

The purification and kinetic analyses of PTPN9 and DUSP9 (Ser277–581) were performed following methods described in the literature, with slight modifications [21]. PAGE analysis data and enzyme kinetic constants are presented in Figure S1 and Table S1, respectively. Enzyme activity in the presence or absence of the compounds was measured using DiFMUP, a representative PTP fluorogenic substrate [21]. First, the inhibitory effects of compounds **1**–**3** were evaluated using 50 µM PTPN9 and 20 µM DUSP9. There was an increase in the inhibitory activity of compound **1** for both enzymes as the number of CH_2_ units increased from **9** to **14** (Figure 2A,B). Compounds **2** and **3** exhibited greater inhibitory effects against PTPN9 than DUSP9 (Figure 2A,B).

Next, the IC_50_ values of the compounds, which induce a 50% inhibition of DiFMUP hydrolytic enzyme activity, were determined (Table 1). The IC_50_ values were determined from the dose–response enzyme inhibition curves of each compound. The dose–response curves for compounds **1e** and **1f** against PTPN9 and DUSP9, respectively, are presented in Figure 2C,D. Dose–response curves were also obtained for the other compounds (Figures S2 and S3). In addition, the evaluation of the expected octanol/water partition coefficient (logP) values for the compounds showed that compound **1d,** an alkyloxy surrogate of GA, had a lower logP value than GA (Table 1).

While the O-alkyl surrogate of GA (**1d**) exhibited approximately three-fold lower inhibitory effects (IC_50_ = 8.61 μM) against DUSP9 than GA (IC_50_ = 3.64 μM), its inhibitory effects against PTPN9 (IC_50_ = 28.54 μM) were similar to those of GA (IC_50_ = 21.80 μM). Compounds **1e** and **1f** exhibited more significant inhibitory effects against both PTPs than **1d**. Inhibition potency was proportional to the length of the O-alkyl substituent. Compounds **1e** and **1f** exhibited higher inhibitory effects against PTPN9, but lower inhibitory effects against DUSP9 compared to GA. Interestingly, compounds **2** and **3**, which are regioisomers of **1e**, exhibited similar inhibitory effects against DUSP9 and significantly lower inhibitory effects against PTPN9 than **1e** (Table 1). This finding indicated that a substitution at the C3 position is essential for PTPN9 inhibition. In a previous study, we reported that PTPN9 inhibition is more important for increasing cellular glucose uptake than DUSP inhibition [21]. In addition, DUSP9 inhibition is not desirable for antidiabetic drugs. On the basis of the findings of previous studies and the findings of this SAR analysis, **1e** and **1f** were selected as lead compounds for subsequent investigations.

### 2.3. Molecular Docking Analysis of GA and Its Analogs

To further explore the molecular basis of the interactions between GA and PTPs, a docking analysis was performed to investigate the correlation between the length of the O-alkyl group and the inhibitory potency of the compounds against PTPN9 and DUSP9. Both PTPs conserve the signature motifs of their active sites. The catalytic “P-loop” contains the signature HCX5R motif and its nucleophilic cysteine residue; both are essential for coordinating the phosphorylated substrate and catalytic dephosphorylation [5,27]. The WPD loop is another conserved PTP motif. When the substrate binds to PTP, the mobile WPD loop encloses the P-loop, and the catalytically important aspartate residue participates in dephosphorylation [28,29]. Although the catalytic sites of most PTPs are highly conserved, the topology and charge distribution along the surface are extremely diverse among subtypes. These structural features contribute to the substrate specificity of PTPs, making them interesting molecular targets for subtype selectivity design [28].

**Figure 3:**
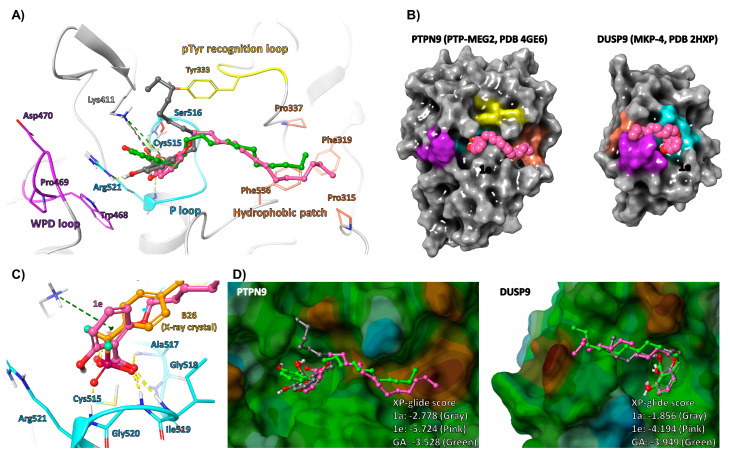
Docking model between GA and its analogs and PTPN9 and DUSP9. (**A**) Binding pose of GA and its analogs on PTPN9. Here, **1a** (gray), **1e** (pink), and GA (green) are presented in a ball-and-stick model. PTPN9 is represented as a series of white ribbons, and the key structure and residues are highlighted. Hydrogen bonds, cation–π interactions, and the salt bridge are represented with yellow, green, and purple dashed lines, respectively. (**B**) Surface representation of PTPN9 and DUSP9. The docking pose for **1e** is represented in CPK. The location of the key structure is highlighted with the corresponding color. (**C**) Superimposition of the docking model of **1e** and X-ray ligand (B24, orange) on P-loop. (**D**) Lipophilic surfaces of PTPN9 and DUSP9 with GA and GA analogs. Blue and brown indicate where hydrophilic and lipophilic features are dominant, respectively.

Within the crystal structure of the PTPN9-inhibitor complex, the 3-iodobenzamide moiety sits above a unique hydrophobic patch formed by the Pro315, Phe319, Pro337, and Phe556 residues and, significantly, interacts via π–π stacking and hydrophobic interactions (Figure 3A) [30]. An inhibitor has been reported to show nanomolar potency against PTPN9 and is highly selective with respect to other PTPs (IC**_50_** > 10 μM). As shown in Figure 1, in the docking model of **1e**, analogs with longer O-alkyl chains (*n* > 11) could reach and interact with the PTPN9 hydrophobic patch (Figure 3B,D). In addition, the docking score for **1a** (*n* = 8) was significantly lower than those for GA and **1e**, indicating that this interaction with the hydrophobic patch is important for the PTPN9 inhibitory effects of GA and its analogs. Furthermore, the 2-hydroxybenzoic acid moiety effectively binds to the catalytic P-loop. As compared to the complexed ligand in the X-ray structure, the carboxylate group of **1e** aligned well with the pTyr mimetic phosphonodifluoromethyl phenylalanine moiety (Figure 3C). Multiple hydrogen bonds were formed between the residues of the P-loop (Cys515 to Ile519) and the carboxylate group of **1e**; cation–π interactions between the Lys411 and phenyl groups enhance the binding potential to the 2-hydroxybenzoic acid derivatives.

DUSP9 also has a smaller hydrophobic patch composed of Pro238, Ile255, Pro256, and Phe268 at a site opposite PTPN9. As with PTPN9, long O-alkyl chains align with this hydrophobic patch, while short analogs cannot reach the hydrophobic site. Our docking model suggested that GA and its analogs could inhibit PTPN9 and DUSP9 by binding with the P-loop and unique hydrophobic patches. The 2-hydroxybenzoic acid moiety of compound **1e** interacted with the catalytic cysteine residues of PTP and could surrogate phosphotyrosine mimetics at a micromolar level. SAR and docking models clearly established correlations between O-alkyl chain length and inhibitory activity.

### 2.4. Compound 1e Increases Glucose Uptake in Mature 3T3-L1 Adipocytes and C2C12 Myotubes

PTPN9 inhibitors have been reported to increase glucose uptake [21]. To clarify the antidiabetic effects of GA analogs, a 2-NBDG (a fluorescent glucose probe) uptake assay was performed using differentiated 3T3-L1 adipocytes and C2C12 myotubes. After 16 h of starvation, differentiated 3T3-L1 adipocytes were incubated with compounds **1e** and **1f** for another 1 h. The cells were subsequently incubated with 2-NBDG for 30 min and washed with PBS before quantifying the fluorescence of 2-NBDG taken up by the cells. The 2-NBDG uptake-increasing effects of GA and its analogs in 3T3-L1 cells were monitored via confocal microscopy.

**Figure 4:**
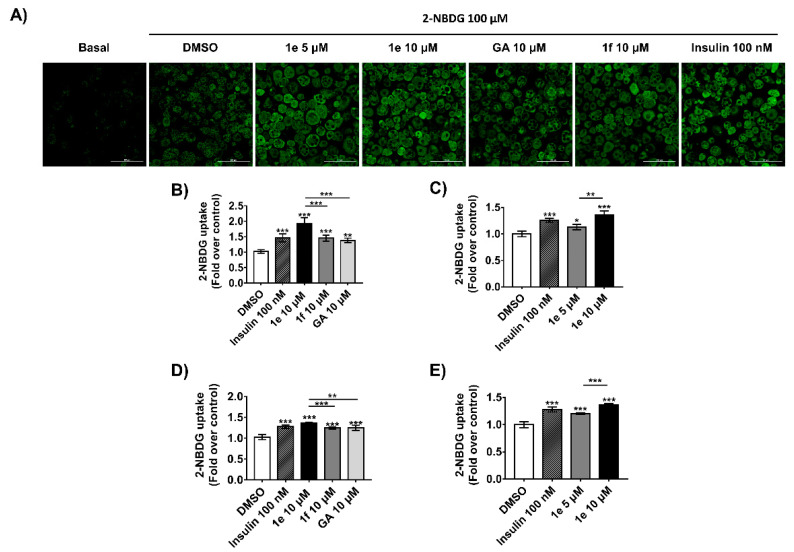
GA and its analogs improved glucose uptake in differentiated 3T3-L1 adipocytes and C2C12 myotubes. After differentiation, mature 3T3-L1 adipocytes were incubated with 0.1% DMSO (the control for basal fluorescence) and 100 nM insulin (the positive control), with GA (10 µM), **1e** (5 or 10 µM), or **1f** (10 µM) as the test compounds in glucose-depleted media supplemented with 0.1% DMSO. Glucose uptake was observed using 2-NBDG as a fluorescence indicator. (**A**) Representative images were obtained by confocal microscopy. (**B**–**E**) For quantification, 3T3-L1 adipocytes (**B**,**C**) or C2C12 myotubes (**D**,**E**) were treated with 2-NBDG, and fluorescence was measured using a plate reader. Data are presented as mean ± standard deviation (*n* = 3). * *p* ≤ 0.05, ** *p* ≤ 0.01, and *** *p* ≤ 0.001 vs. Control DMSO. Statistical analysis was performed using a one-way ANOVA for multiple comparisons, followed by Tukey’s test.

The blank group was set in the absence of 2-NBDG but was treated with DMSO to determine the basal fluorescence levels in 3T3-L1 adipocytes (Figure 4A, Basal); in the negative control group, 3T3-L1 adipocytes were treated with 2-NBDG and DMSO for the evaluation of basal glucose uptake levels (Figure 4A, DMSO). Insulin (100 nM), which was employed as a positive control, also significantly stimulated 2-NBDG uptake, proving the credibility of our experiment. As illustrated in Figure 4, **1e** exhibited the most intense fluorescence, suggesting that it might be the most effective compound for enhancing glucose uptake in 3T3-L1 adipocytes. Furthermore, to quantify 2-NBDG uptake, fluorescence intensity (Ex/Em = 485/535 nm) was measured using a plate reader. As compared to the DMSO control at a concentration of 10 µM, **1e** significantly increased 2-NBDG uptake in both 3T3-L1 adipocytes (Figure 4B) and C2C12 myotubes (Figure 4D) by approximately 2.0 and 1.4 fold, respectively (Figure 4).

### 2.5. Compound 1e Activates Ampk in 3t3-L1 Adipocytes

**Figure 5:**
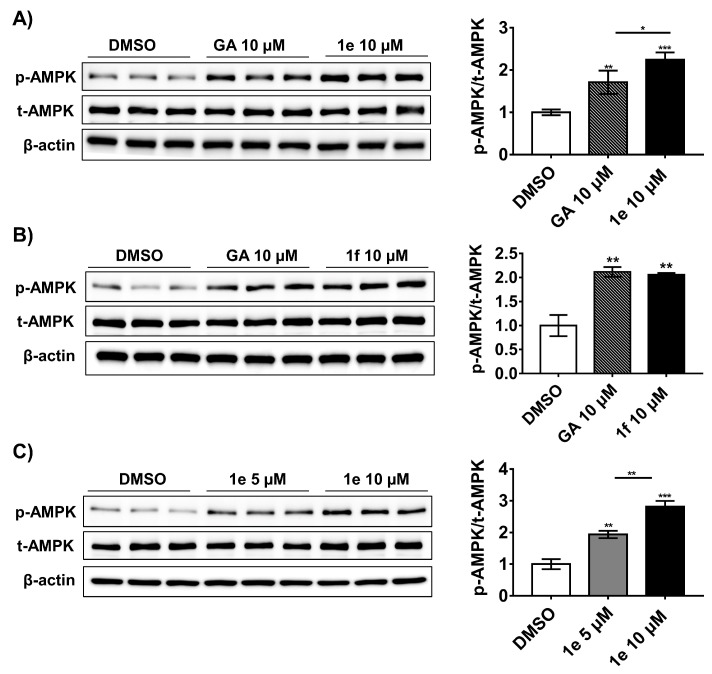
Compound **1e** increased AMPK activation in mature 3T3-L1 adipocytes. (**A**,**B**) Immunoblot analysis of p-AMPK levels in 3T3-L1 adipocytes following treatment with GA, **1e**, and **1f** at 10 µM (**C**). Immunoblot analysis of p-AMPK levels in 3T3-L1 adipocytes following treatment with compound **1e** at the indicated concentrations. The densities of the corresponding bands were quantified using the ATTO CS Analyzer 4 and normalized to that of t-AMPK. Data are presented as mean ± standard deviation (*n* = 3). * *p* ≤ 0.05, ** *p* ≤ 0.01, and *** *p* ≤ 0.001 vs. Control DMSO. Statistical analysis was performed using a one-way ANOVA for multiple comparisons, followed by Tukey’s test.

To further elucidate the pathway involved in **1e**-mediated glucose uptake promotion, AMPK and Akt phosphorylation in mature 3T3-L1 adipocytes was evaluated via Western blotting following treatment with 5 or 10 µM **1e** for 1 h. As compared to GA and **1f, 1e** significantly induced AMPK phosphorylation in a concentration-dependent manner (Figure 5). However, no significant change in Akt phosphorylation levels (*p* < 0.05) was observed in the “compound only” group and insulin co-treatment group (Figure S4), suggesting that **1e**-induced glucose uptake in 3T3-L1 adipocytes involves AMPK activation but not Akt activation.

### 2.6. Compound 1e Attenuates Palmitic Acid (PA)-Induced Insulin Resistance in C2C12 Muscle Cells via the Insulin-Dependent Akt Pathway

**Figure 6:**
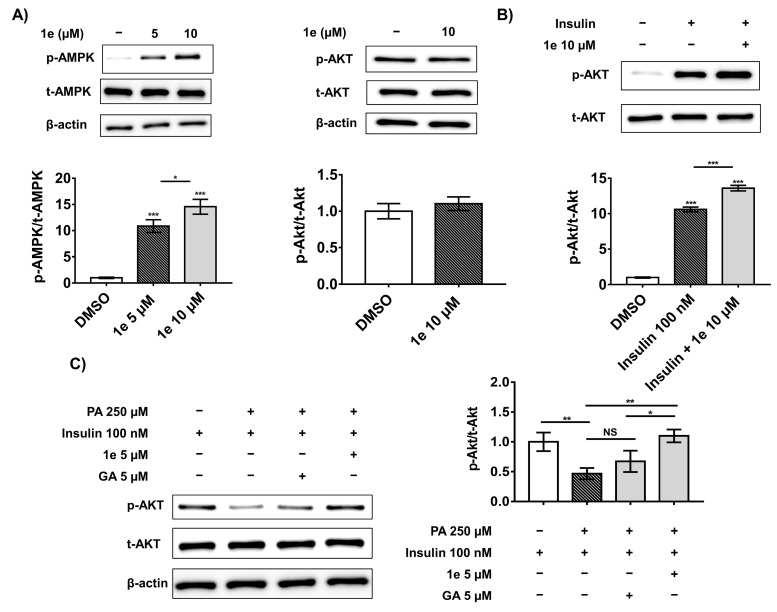
Compound **1e** upregulates p-AMPK levels and exhibits protective effects against PA-induced insulin resistance in C2C12 myotubes. (**A**) Immunoblot analysis of p-AMPK and p-Akt levels in C2C12 myotubes following treatment with **1e** at the indicated concentrations. (**B**) Immunoblot analysis of p-Akt levels following treatment with **1e** at the indicated concentration in the presence or absence of 100 nM insulin. (**C**) Compound **1e** ameliorates PA-induced insulin resistance in C2C12 muscle cells. Differentiated C2C12 myotubes were pre-treated with 250 μM palmitic acid for 16 h and then incubated with 5 μM GA or **1e** for 30 min in the presence of 100 nM insulin. The densities of the corresponding bands were quantified using the ATTO image analysis software (CS analyzer 4) and normalized to that of t-AMPK or t-Akt. Data are presented as mean ± standard deviation (*n* = 3). * *p* ≤ 0.05, ** *p* ≤ 0.01, and *** *p* ≤ 0.001 vs. Control DMSO. Statistical analysis was performed using a one-way ANOVA for multiple comparisons, followed by Tukey’s test.

To further investigate the mechanism of action of **1e**, we evaluated its effects on AMPK and AKT phosphorylation in C2C12 myotubes. As shown in Figure 6A, **1e** significantly upregulated AMPK phosphorylation in a concentration-dependent manner. In line with the results obtained in 3T3-L1 cells, in the absence of insulin, **1e** had no effects on basal p-Akt expression levels (Figure 6A).

Since insulin plays an important role in regulating glucose metabolism through the redistribution of GLUT4 from intracellular vesicles to the cell surface, it is important to describe the interaction between **1e** and insulin in the Akt pathway. Following co-treatment with insulin, **1e** exhibited synergistic Akt phosphorylation-improving effects with insulin (Figure 6B). Next, we evaluated the protective effects of **1e** against PA-induced insulin resistance in C2C12 myotubes. To generate an insulin resistance model, C2C12 myotubes were treated with 250 µM PA in a serum-free medium for 16 h. Akt phosphorylation levels in PA-treated C2C12 cells decreased by 50% compared to levels in normal C2C12 cells (Figure 6C). Furthermore, in the presence of insulin and GA, Akt phosphorylation levels decreased only by 30% compared to levels in normal cells. However, co-treatment with insulin and **1e** ameliorated Akt phosphorylation in PA-induced insulin-resistant C2C12 cells, restoring it to levels similar to those in the non-insulin-resistant group (Figure 6C). In addition, the GA analogs induced no cytotoxic effects in C2C12 muscle cells (data not shown). Collectively, these data suggested that **1e** ameliorated PA-induced insulin resistance in C2C12 muscle cells via the insulin-dependent Akt pathway more effectively than GA.

### 2.7. Compound 1e Improves Glucose Uptake by Inhibiting PTPN9 without Activating other Related Receptors

**Figure 7:**
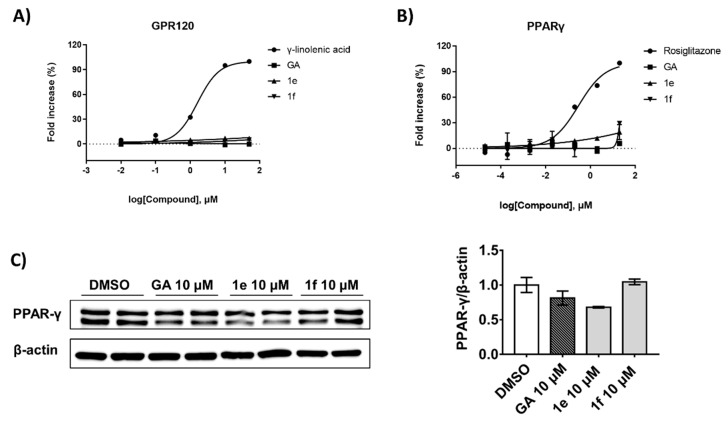
Target selectivity of **1e**. (**A**,**B**) GA analogs induced GPR120 and PPARγ transcriptional activity. Transcriptional activity was determined in CHO cells transiently co-transfected with the GPR120 plasmid and the luciferase reporter, SRE-Luc (**A**), or with PPARγ and PPRE-Luc (**B**). Three hours following transfection, the cells were treated with indicated concentrations of the compounds for an additional twenty−four hours. Here, γ-linolenic acid (**A**) or rosiglitazone (**B**) was used as the positive control. (**C**) Immunoblot analysis of PPARγ levels in 3T3-L1 adipocytes following treatment with GA and **1e** at the indicated concentrations. The densities of corresponding bands were quantified using the ATTO CS Analyzer 4 and normalized to that of β-actin. Data are presented as mean ± standard deviation (*n* = 2).

Glucose uptake is regulated by three major mechanisms. First, through the activation of the Akt pathway, which promotes GLUT4 translocation via the activation of the signaling cascade involving the insulin receptor substrate (IRS) family and PI3 kinase through tyrosine phosphatases or G-protein-coupled receptors (GPCRs) [31]; second, through the upregulation of AMPK phosphorylation, which increases glucose uptake by inducing GLUT4 translocation to the plasma membrane [32,33]; third, through the promotion of GLUT4 expression by PPARγ ligands [34]. In this study, we demonstrated that GA analogs increased AKT and AMPK phosphorylation through the inhibition of PTPN9. However, these compounds may induce GLUT4 expression and improve insulin sensitivity in the treated cells through their interactions with GPCR and/or PPARγ. Among GPCRs highly expressed in 3T3-L1 adipocytes and C2C12 muscle cells, GPR120 is the receptor that is most closely associated with glucose uptake [35,36]. Hence, to determine if GA analogs increased glucose uptake via interactions with GPR120 and/or PPARγ, they (**1e** and **1f**) were subjected to a reporter gene assay at a concentration of 50 μM using CHO cells transiently co-transfected with the GPR120 plasmid and luciferase reporter, SRE-Luc (Figure 7A), or with PPARγ and PPRE-Luc (Figure 7B,C). No significant change was observed, indicating that **1e**-induced glucose uptake was mediated by PTPN9 and DUSP9 inhibition without the activation of other related receptors.

## 3. Discussion

Type 2 diabetes mellitus (T2DM) is a progressive metabolic disorder characterized by elevated circulating glucose due to glucose intolerance, caused by insufficient insulin production from the pancreas, and resistance to insulin action. It constitutes a serious health burden that affects 451 million adults worldwide as of 2017, and this number is expected to increase to 693 million by 2045 [3]. The practical approach to managing patients includes the initiation of medical treatments involving biguanides, sulfonylureas, and thiazolidinediones (TZD), usually along with the risk of heart failure and hypoglycemia, weight gain, and renal impairment [37]. As such, many pharmaceutical companies and research institutes in the world have invested in new and safe anti-T2DM targets in the hope of producing better-tolerated antidiabetic drugs [38].

Skeletal muscle cells and adipocytes play a pivotal role in glucose uptake, insulin resistance, and hyperglycemia [39,40]. In the skeletal muscle and adipose tissue, AMPK activation improves GLUT4 translocation and further increases glucose uptake [25]. Thus, compounds that increase glucose uptake via AMPK activation would be good candidates for T2DM. In our previous study, we had verified that PTPN9 and DUSP9 knockdown induces an upregulation in the phosphorylation of AMPK. Compared with DUSP9, PTPN9 knockdown resulted in a greater increase in AMPK phosphorylation, while Brice Emanuelli showed that DUSP9 has a protective effect on insulin resistance by dephosphorylating EAK and JNK, which have been shown to induce insulin resistance [41]. Therefore, compounds with significant PTPN9 inhibitory effects and moderate or no DUSP9 inhibitory effects would be more favorite as antidiabetic drug candidates.

Recently, we reported the bioactivity of a novel PTPN9 inhibitor, ginkgolic acid (C13:0), stimulated glucose uptake in 3T3L-1 and C2C12 cells through the activation of the AMPK signaling pathway. In this study, aiming to facilitate the convenient synthesis of GA analogs, we synthesized a series of GA alkoxy surrogates and evaluated their SAR with respect to antidiabetic effects. The SAR analysis revealed that efficient PTPN9 inhibition required a substitution with an alkyl chain equal in length or longer than GA at the 6-position of salicylic acid (compound **1**). This finding suggested that compared to alkyl analogs, GA alkoxy surrogates proved to be a more efficient way to discover new hits because of the lower synthesis difficulty and the higher yield.

After conducting the enzymatic assay, **1e** and **1f** were selected as hit compounds that exhibited a better inhibitory effect against PTPN9 with IC_50_ values of 10.20 ± 0.52 μM and 18.31 ± 0.17 μM, respectively, which were more than GA (21.80 ± 0.45 μM). Although **1e** showed fewer significant inhibitory effects against PTPN9 than **1f**, **1e** promoted glucose uptake in 3T3-L1 adipocytes and C2C12 myotubes more significantly than **1f** and GA. We concluded that is because of the lipophilicity of both compounds, and the LogP value for **1e** (6.07) was found to fall between that of **1f** (6.91) and GA (6.70). The high hydrophobicity of **1e** may have led to a decrease in its cellular uptake.

In our mechanism study, **1e** treatment activated AMPK independently of insulin in 3T3-L1 adipocytes. However, **1e** stimulates insulin sensitivity, which is coupled to the basal glucose translocation of GLUT4, and attenuates palmitate-induced insulin resistance in C2C12 muscle cells via the insulin-dependent Akt pathway. As mentioned above, one of the major mechanisms of glucose uptake is through the activation of the Akt pathway via the activation of the signaling cascade involving the insulin receptor substrate (IRS) family and PI3 kinase through tyrosine phosphatases or G-protein-coupled receptors (GPCRs). However, the recruitment of PI3 kinase through G-protein-coupled receptors (GPCRs) is performed in an insulin-independent manner. Since in the absence of insulin, **1e** exhibited no significant effect in Akt phosphorylation, we demonstrated that **1e** increases glucose uptake, but not by activating the associated GPCRs. This finding is consistent with the reporter gene assay, wherein **1e** improved glucose uptake by inhibiting PTPN9 without activating other related receptors (Figure S5).

In summary, **1e** was selected as a strong PTPN9 inhibitor and moderate DUSP9 inhibitor for antidiabetic effect evaluation. We found that **1e** increases glucose uptake in mature 3T3-L1 adipocytes and C2C12 myotubes and ameliorated palmitate-induced insulin resistance in C2C12 cells more significantly than GA via the insulin-dependent Akt pathway. Collectively, **1e** may serve as a novel scaffold for developing more potent PTPN9 inhibitors with significant potency and molecular properties suitable for the further development of novel antidiabetic agents.

## 4. Materials and Methods

### 4.1. Overexpression and Purification of Recombinant PTPN9 and DUSP9

The methods employed for PTP overexpression and purification have been described in a previous study [21]. In brief, human *PTPN9* and *DUSP9* were cloned into a pET28a vector (Merk Millipore, Darmstadt, Germany) containing a hexahistidine (His6) tag or a maltose-binding protein (MBP) tag in its N-terminus (see Supplementary Materials). Recombinant genes were separately transformed into *Escherichia coli* Rosetta (DE3) (Merk Millipore, Darmstadt, Germany), and the overexpression of each gene was induced via the addition of 0.1 mM IPTG (Ducherfa Biochemie, Haarlem, RV, USA). After 16 h of incubation at 18 °C, cell pellets were obtained via centrifugation (3570× *g* at 4 °C for 15 min), resuspended in lysis buffer (50 mM Tris, pH: 7.5, 500 mM NaCl, 5% glycerol, 0.025% 2-mercaptoethanol, and 1 mM phenyl-methylsulfonyl fluoride (PMSF)), and lysed by ultrasonication. Subsequently, cell lysates were centrifuged at 29,820× *g* for 30 min at 4 °C; then, the supernatant was collected and incubated with a cobalt affinity resin (TALON^®^ Metal Affinity Resin, Takara Korea, Seoul, Korea). After 1 h of incubation at 4 °C, the desired proteins were eluted by adding the lysis buffer supplemented with 100 mM imidazole. Samples were stored at –80 °C until use.

### 4.2. Half-Maximal Inhibitory Concentration (IC_50_) Determination

The enzymatic activities of purified PTPN9 and DUSP9 were measured using 6,8-difluoro-4-methylumbelliferyl phosphate (DiFMUP), the most widely used fluorogenic PTP substrate in the literature [42]. To determine *K*_M_ values, PTPN9 (0.15 nM) and DUSP9 (200 nM) were added to a reaction buffer (20 mM Bis-tris, pH: 7.0, 150 mM NaCl, 2.5 mM dithiothreitol (DTT), and 0.01% Triton X-100) containing DiFMUP (800, 400, 200, 100, 50, 25, 12.5, or 6.25 μM) in a 96-well black plate. The final reaction volume was 100 µL. The fluorescence intensity was continuously monitored for 10 min (Ex/Em = 355/460 nm) on a Victor™ X4 multilabel plate reader (Perkin Elmer, Norwalk, CT, USA). *K*_M_ values were obtained using Lineweaver–Burk plots analysis.

To evaluate the enzyme inhibitory effects of GA and its analogs, 10 µl of the enzyme stock solution (1.5 nM PTPN9 and 2 µM DUSP9) in the reaction buffer was added into the mixture of DiFMUP (to a final concentration of 220 µM and 90 µM for PTPN9 and DUSP9, respectively) and each compound (to a final concentration of 50 µM and 20 µM for PTPN9 and DUSP9, respectively) was added to the reaction buffer. Enzyme activity was measured as described above. Half-inhibitory concentration (IC_50_) values were determined under similar conditions, except for the concentrations of the inhibitors (200, 100, 50, 25, 12.5, 6.25, or 3.125 µM for PTPN9 and 25, 12.5, 6.25, 3.125, 1.563, 0.782, or 0.391 µM for DUSP9). The dose-dependent curve was fitted to the four-parameter logistic regression equation, (y = a + (b – a)/(1 + (x/c)^d^), to obtain IC_50_ values using the Kaleida Graph software Ver. 4.1.3. (Synergy Software Inc, PA, USA) [43].

### 4.3. Molecular Modeling

Ligand docking was carried out using Schrödinger maestro 2020-4 (operated under Windows 10; New York, NY, USA) [44]. Ligands were sketched using a 2D sketcher module or downloaded as .sdf files from the PubChem database (https://pubchem.ncbi.nlm.nih.gov/ accessed on 15 July 2021) and prepared using the Ligprep module. All possible ionized states for the ligands were generated under physiological pH conditions (including the neutral state) and were further used for docking. The X-ray crystal structures of human PTPN9 (PTP-MEG2, PDB ID: 4GE6) and DUSP9 (MKP-4, PDB ID: 2HXP) were downloaded from the RCSB Protein Data Bank (https://www.rcsb.org/ acceded on 6 August 2021) [30,45]. Downloaded receptors were prepared using the Protein preparation module. Missing side chains and loops were filled in using the Prime program, and water molecules, as well as all unnecessary molecules, were removed. The mutated DUSP9 Ser290 residue was restored to Cys290 prior to minimization. The centers of the grid boxes were defined using a peptide PTPN9 inhibitor and a catalytic cysteine residue (Cys290) for DUSP9 in its crystal structure. All grid boxes were set to 25 Å. For docking, XP-glide in the Ligand docking module was used in default settings without constraints. Twenty poses per ligand were exported for manual comparison.

### 4.4. Cell Culture and Differentiation

Mouse 3T3-L1 pre-adipocytes (Zenbio, Durham, NC, USA) were incubated in high glucose Dulbecco’s modified Eagle medium (DMEM; Welgene Biotech Co., Ltd., Gyeongsan-si, Korea) supplemented with 10% bovine calf serum (BCS; Thermo Fisher Scientific Korea Ltd., Seoul, Korea) and 1% 100× antibiotic–antimycotic solution (Welgene Biotech Co., Ltd., Gyeongsan-si, Korea) at 37 °C in 5% CO_2_. All the reagents in this section were at their final concentration. After 3T3-L1 cells reached 100% confluence, differentiation was induced by the addition of DMEM supplemented with 10% fetal bovine serum (FBS; Welgene Biotech Co., Ltd., Gyeongsan-si, Korea), 1% 100× antibiotic–antimycotic solution, 0.5 mM isobutylmethylxanthine (IBMX; Merk KGaA, Darmstadt, Germany), 1 µM dexamethasone (Sigma-Aldrich, Saint Louis, MS, USA), and 5 µg/mL insulin (Merck KgaA, Darmstadt, Germany), followed by incubation for 48 h at 37 °C. Then, the cells were incubated in DMEM supplemented with 10% fetal bovine serum (FBS; Welgene Biotech Co., Ltd., Gyeongsan-si, Korea), 1% 100× antibiotic–antimycotic solution, and 5 μg/mL insulin (differentiation medium II) for another 48 h, and further cultured in DMEM containing only 10% fetal bovine serum (FBS; Welgene Biotech Co., Ltd., Gyeongsan-si, Korea) and 1% 100× antibiotic–antimycotic solution; this was replaced with a fresh medium every 48 h. Mouse C2C12 myoblasts (ATCC, Manassas, VA, USA) were grown to 90% confluence in DMEM supplemented with 20% FBS and 1% 100× antibiotic–antimycotic solution; then, differentiation was induced by the addition of 2% horse serum in DMEM, with incubation for 4 days. The medium was replenished every 2 days. Mouse C2C12 and 3T3-L1 cells were maintained at 37 °C in 5% CO_2_. CHO cells were maintained in DMEM (Gibco BRL, Thermo Fisher Scientific Korea Ltd., Seoul, Korea) containing 10% heat-inactivated FBS, 4.5 g/L glucose, and 1% penicillin (HyClone™, Cytivalifescience, Seoul, Korea) at 37 °C in 5% CO_2_. MIN6 cells were maintained in DMEM containing 15% heat-inactivated FBS, 4.5 g/L glucose, and 1% penicillin at 37 °C in 5% CO_2_.

### 4.5. Western Blotting

The expression levels of glucose uptake-related proteins were determined via Western blotting, as previously described [46]. In brief, differentiated 3T3-L1 cells were plated in six-well plates at a 1.5 × 10^4^ cells/well density in a final volume of 2 mL. Then, the cells were incubated in the presence of DMSO or different concentrations of the compounds and harvested using a RIPA buffer (Sigma-Aldrich, Saint Louis, MS, USA) and a protease inhibitor cocktail (Roche Korea, Seoul, Korea) after 1 h of incubation. Equivalent amounts of proteins (20 µg) from each lysate were separated by 10% SDS-PAGE and transferred onto PVDF membranes (Merk KgaA, Darmstadt, Germany), where they were maintained for 120 min at 200 mA. The membranes were blocked with 5% skim milk for 1 h at room temperature in 0.1% TBST and then probed with primary antibodies at different concentrations in 0.1% BSA overnight at 4 °C according to the manufacturer’s instructions. Secondary antibodies were added to the membranes at a concentration of 1:2000 for 1 h at room temperature, and immunoreactive bands were detected via ECL (GE Healthcare Korea Co., Ltd., Incheon, Korea). The primary antibodies used were anti-p-AMPK (cat.# 2535), anti-t-AMPK (cat.# 2532), anti-p-Akt (cat.# 4060), anti-t-Akt (cat.# 4691), anti-PPARγ (cat.# 2430) (Cell Signaling Technology, Inc., Beverly, MA, USA), and anti-beta-actin (AB Frontier Co., Ltd., Seoul, Korea) antibodies.

### 4.6. Glucose Uptake Assay

Glucose uptake assays were performed following previously described methods with slight modifications [47]. In brief, mature 3T3-L1 and C2C12 cells were starved in low-glucose DMEM (Gibco BRL, Thermo Fisher Scientific Korea Ltd., Waltham, MA, USA) for 16 h and then incubated for 1 h with 10 µM GA, **1e**, or **1f**, or for 30 min with 100 nM insulin in glucose-depleted DMEM (Gibco BRL, Thermo Fisher Scientific Korea Ltd., Waltham, MA, USA). Subsequently, 100 µM 2-[N-(7-nitrobenz-2-oxa-1,3-diazol-4-yl) amino]-2-deoxyglucose (2-NBDG; Thermo Fisher Scientific Korea Ltd., Seoul, Korea) was added to the cells, which were then incubated at 37 °C for 1 h. After incubation, the cells were washed with pre-cold PBS, and the fluorescence intensity was measured using a fluorescence microplate reader (Victor^TM^ X4, PerkinElmer, Waltham, MA, USA) (Ex/Em = 485/535 nm). For cell imaging, 3T3-L1 pre-adipocytes were seeded into µ-Slide 8 wells (Ibidi, München, Germany), and their differentiation was induced. Mature 3T3-L1 adipocytes and C2C12 myotubes were prepared as described earlier. Glucose uptake images were obtained at Ex/Em = 488/516 nm using a confocal microscope (LSM700 laser scanning confocal microscope, Carl Zeiss, Oberkochen, Germany).

### 4.7. Palmitate-Induced Insulin Resistance in C2C12 Myotubes

The preparation of PA–BSA conjugates was carried out following a previously described method with slight modifications [48]. In brief, to prepare 7.5 mM PA–BSA conjugates (1:5 molar ratio), sodium palmitate (Sigma-Aldrich, Saint Louis, MS, USA) was dissolved in 150 mM NaCl at 70 °C by adding it to 150 mM NaCl containing 10% (*w*/*v*) fatty acid-free BSA at 30 °C at a pH of 7.4 with stirring; this was followed by filtration (0.22 µm). Differentiated C2C12 cells were starved in serum-free DMEM for 3 h. Then, insulin resistance was induced by incubating the cells with 250 µM PA–BSA conjugate in DMEM for 16 h.

### 4.8. Reporter Gene Assay

This was performed following a previously described method [49]. In brief, CHO cells were plated to 96-well white plates (REF3610, Corning Life Sciences, NY, USA) in DMEM (HyClone^TM^, Cytivalifescience, Seoul, Korea) supplemented with 10% FBS and 1% penicillin at a density of 1.5 × 10^4^ cells/well. After 24 h of incubation, the cells were co-transfected using the Cignal PPAR Reporter (QIAGEN, Cignal PPAR (Luc) Reporter kit, CCS-3026L) and pCMV6-hFFAR4-GFP (RG201538, OriGene Technologies, Inc., Rockville, MD, USA) or with SRE plasmids (pGL4.33 (luc2P/SRE/Hygro), Promega, Madison, WI, USA) and FFAR4 (RG231184, OriGene Technologies, Inc., Rockville, MD, USA) using a Lipofetamine 3000 device (Invitrogen, Thermo Fisher Scientific Korea, Ltd., Seoul, Korea) according to the manufacturer’s instructions. After 6 h of incubation, the transfected CHO cells were treated with various concentrations of the compounds or the positive coFtablntrol. Control cells were treated with 0.1% DMSO. After incubation for another 24 h, luciferase activity was measured using the dual-luciferase assay system (Promega Korea, Seoul, Korea) according to the manufacturer’s instructions. All the individual in vitro assays to determine the average efficiency were performed twice and in triplicate for each treatment.

### 4.9. Statistical Analysis

To identify significant differences, data were analyzed using GraphPad Prism 7 (GraphPad Software, Inc., San Diego, CA, USA). After testing the normality of distribution using a Shapiro–Wilk test at α = 0.05, statistical significance (*p* < 0.05) was assessed using a two-tailed unpaired t-test for comparisons between two groups and a one-way ANOVA was used for multiple comparisons, followed by Tukey’s test. (GraphPad Software, San Diego, CA, USA). Data are represented as the mean ± standard deviation (S.D.).

## Figures and Tables

**Table 1:** The activity of the GA analogs.

Compound	Structure	Partition Coefficient (log *p*) ^a^	IC_50_ (μM) (PTPN9) ^b^	IC_50_ (μM) (DUSP9) ^c^
GA	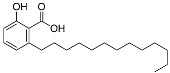	6.7	21.80 ± 0.45	3.64 ± 0.11
**1a**	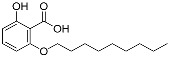	4.4	NA	NA
**1b**	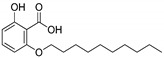	4.82	NA	NA
**1c**	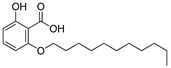	5.24	77.09 ± 0.78	23.85 ± 0.65
**1d**	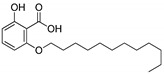	5.65	28.54 ± 0.1	8.61 ± 0.66
**1e**	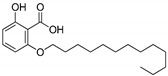	6.07	18.31 ± 0.17	6.40 ± 0.87
**1f**	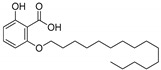	6.91	10.20 ± 0.52	5.87 ± 0.55
**2**	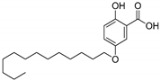	6.07	69.28 ± 6.22	5.27 ± 0.07
**3**	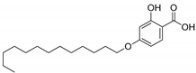	6.07	36.52 ± 1.00	4.68 ± 0.06

NA: no activity; ^a^ logP values were calculated using BioByte’s algorithm in ChemBioDraw Ultra 14.0; ^b^ the IC50 values for PTPN9 inhibitory activity represent the mean of three experiments; ^c^ the IC50 values for DUSP9 inhibitory activity represent the mean of three experiments. The related graph for calculating IC_50_ were presented in Figures S2 and S3. Data are presented as mean ± standard deviation (*n* = 2).

## Data Availability

All the data are provided in the manuscript. Detailed methods and additional data are available upon request from the corresponding authors.

## Supplementary Materials

The following supporting information can be downloaded at: https://www.mdpi.com/article/10.3390/ijms23073927/s1.
