# Supplementary material for: Structure–Activity Relationship of Synthetic Ginkgolic Acid Analogs for Treating Type 2 Diabetes by PTPN9 Inhibition
Source: Int J Mol Sci. 2022 Apr 1;23(7):3927. doi: 10.3390/ijms23073927 (PMC8999917; doi:10.3390/ijms23073927)
Supplement: Supplementary file 1 [file ijms-23-03927-s001.zip › ijms-1655950-supplementary.pdf]

Supplementary Materials

Structure–Activity Relationship of Synthetic Ginkgolic Acid Analogs for Treating Type 2 Diabetes by PTPN9 Inhibition

Jinsoo Kim [1,†], Jinyoung Son [2,†], Dohee Ahn [1], Gibeom Nam [1], Xiaodi Zhao [1], Hyuna Park [2], Woojoo Jeong [2] and Sang J. Chung [1,2,*]

[1] School of Pharmacy, Sungkyunkwan University, Suwon 16419, Korea; neto543@naver.com (J.K.); ehgml94@naver.com (D.A.); skarlqja12@skku.edu (G.N.); zhaoxiaodi1019@gmail.com (X.Z.)

[2] Department of Biopharmaceutical Convergence, Sungkyunkwan University; Suwon 16419, Korea; qoxmfspt753@naver.com (J.S.); hyunaaa_1226@naver.com (H.P.); jwjoo5@naver.com (W.J.)

* Correspondence: sjchung@skku.edu

† These authors contributed equally to this work.

Table of Contents

Table S1. Kinetics constants of PTPN9 and DUSP9. 2

Figure S1. The purified gel of DUSP9 and PTPN9. 3

Figure S2. IC_{50} values of compounds against PTPN9. 4

Figure S3. IC_{50} Values of compounds against DUSP9. 5

Figure S4. Effect of GA, 1e, and 1f on AKT activation. 6

Figure S5. Signaling pathways studied in 3T3-L1 adipocytes and C2C12 myotubes. 7

Experimental procedure for the synthesis of GA analogues....................................8

Spectral data and HPLC chromatogram....................................14

Table S1. Kinetics constants for DiFMUP hydrolysis by PTPN9 and DUSP9.

	[E] (nM)	K_m (μM)	V_{max} (μMmin^{-1})	k_{cat} (min^{-1})	k_{cat} /K_m (μM^{-1}min^{-1})
PTPN9	0.15	110	1.73	1.2×10^4	109.1
DUSP9	200.0	45	1.65	8.25	0.2

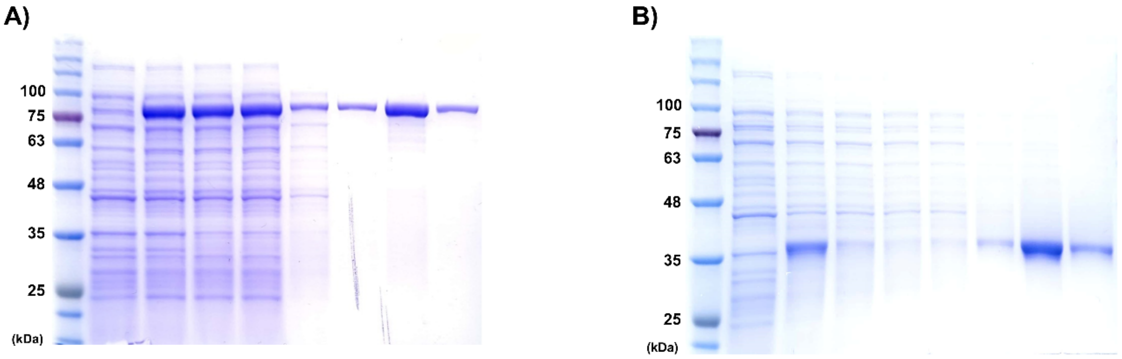

Figure S1. The purified gel of DUSP9 (uniprot number: Q99956) and PTPN9 (uniprot number: P43378). (**A**) DUSP9 full sequence was purified (86.8 kDa); (**B**) PTPN9 from Ser277 to Ser581 was purified (37.6 kDa).

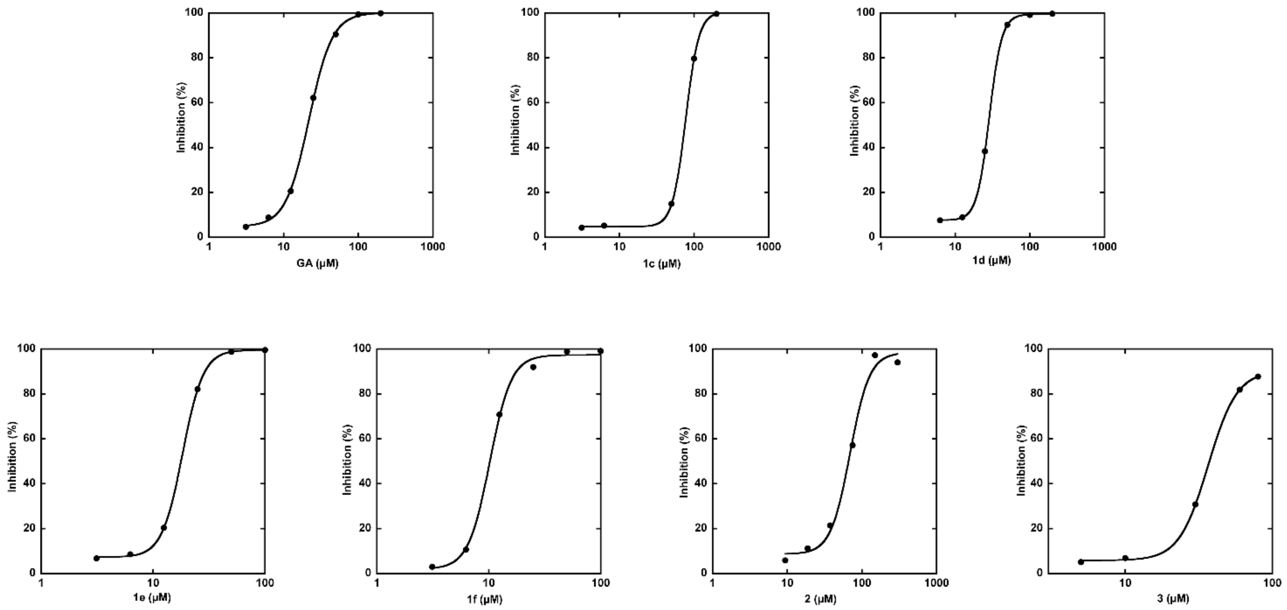

Figure S2. IC_{50} values of compounds against PTPN9.

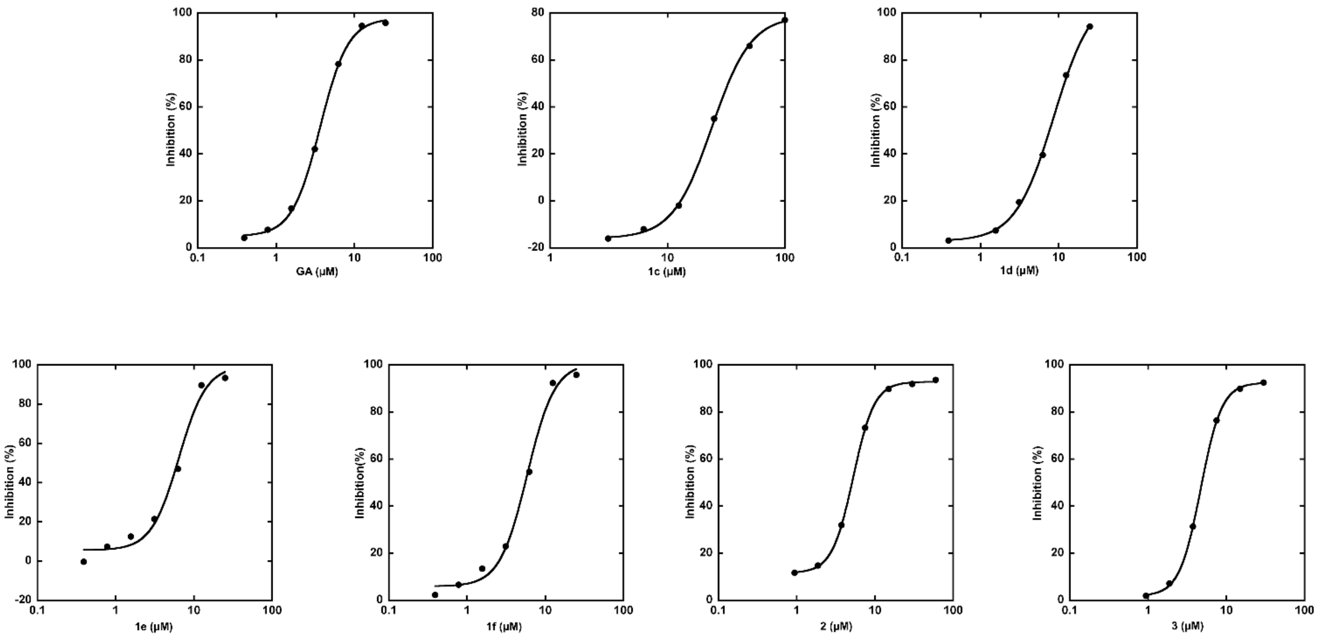

Figure S3. IC_{50} Values of compounds against DUSP9.

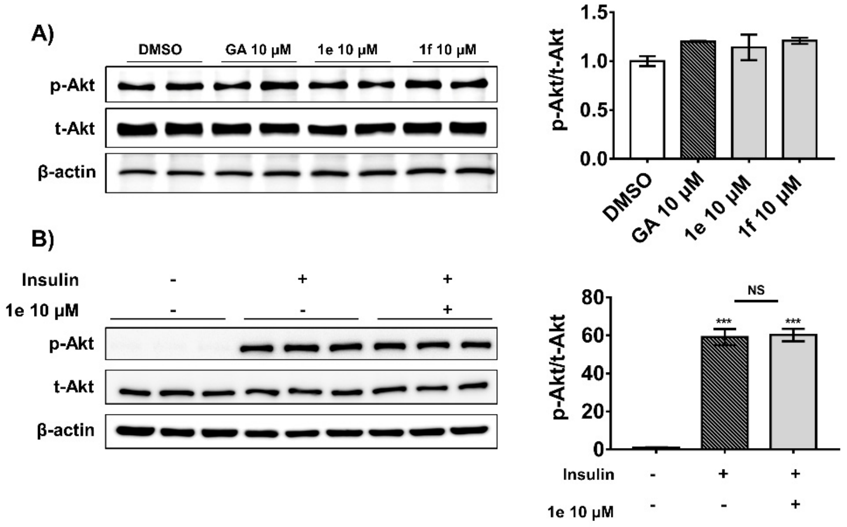

Figure S4. Effect of GA, 1e, and 1f on AKT activation. Immunoblot analysis of p-Akt levels was performed following treatment with GA and its analogs at 10 μM in the absence (**A**) or presence (**B**) of 100 nM insulin. The densities of the corresponding bands were quantified using the ATTO CS Analyzer 4 and normalized to that of t-Akt. Data are presented as mean ± standard deviation (n = 3). ***P ≤ 0.001 vs. Control DMSO. NS: no significance. Statistical analysis was performed by one-way ANOVA for multiple comparisons followed by Tukey's test.

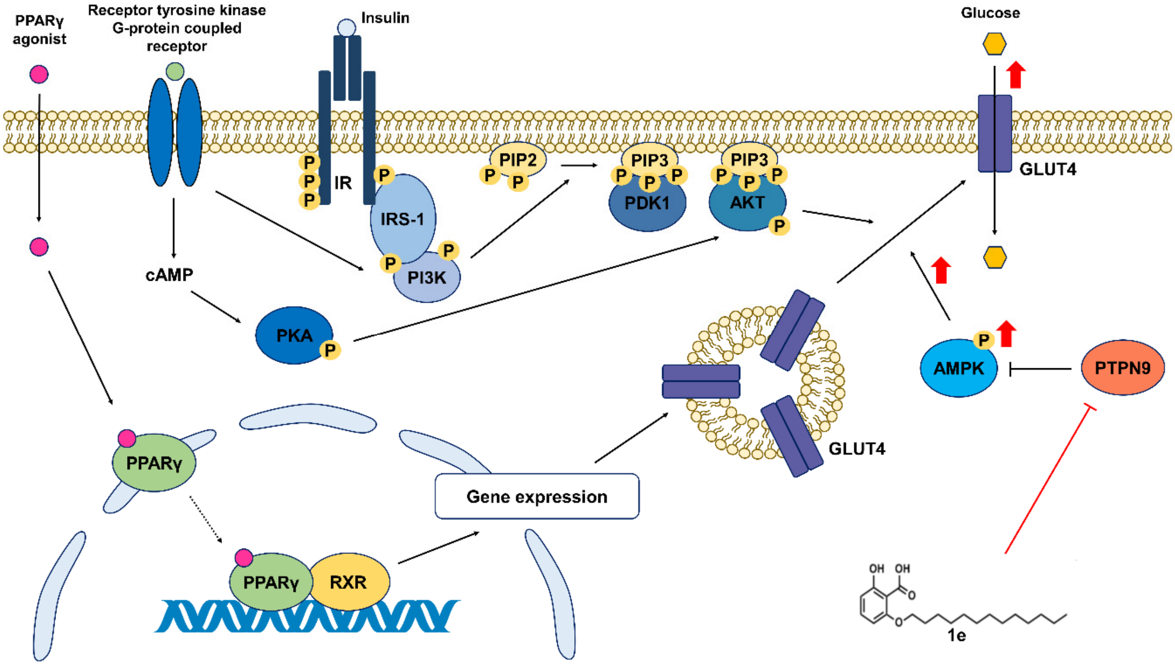

Figure S5. Signaling pathways studied in 3T3-L1 adipocytes and C2C12 myotubes.

Experimental procedure for the synthesis of GA analogues

General Information

Unless otherwise noted, all reagents were purchased from Sigma-Aldrich (Gangnamgu, Seoul, Korea). The series of 1-bromoalkane were purchased Tokyo Chemical Industry (Yangcheongu, Seoul, Korea). All solvents were purchased from DaeJung Chmicals & Metals (Siheung, Korea).

^{1}H NMR was recorded using a Varian Oxford 300 MHz NMR spectrometer (Agilent Technologies, Santa Clara, California, USA) and ^{13}C NMR was recorded using a Avance III 700 NMR spectrometer (Bruker Corporation, Billerica, Massachusetts, USA) using TMS as the internal standard and $CDCl_3$ as the solvent. HPLC analysis was performed on a Waters 2695 separation module (Waters Corporation, Milford, Massachusetts, USA) using a Xbridge C_{18} column (5 μm, 250 × 4.6 mm), at a flow rate of 1.0 mL/min, temperature 25 °C and an injection volume of 10 μL (the eluent was a mixture of 0.1% TFA in water (A) and 0.075% CAN (B)). High-resolution mass spectra were recorded with a JMS-700 MStation (JEOL Ltd., Akishima, Tokyo, Japan) and LC-Mass spectra were recorded with a Shimadzu LCMS-2020 (Shimizu Corporation, Kyobashi, Tokyo, Japan).

Synthesis of 5-hydroxy-2,2-dimethyl-4H-benzo[d][1,3]dioxin-4-one (5).

Under argon atmosphere, acetone (1.26 ml, 16.87 mmol) and $SOCl_2$ (1.22 mL, 16.87 mmol) were added to a solution of 2,6-dihydroxybenzoic acid (**4**) (2.00 g, 12.98 mmol) and DMAP (159.00 mg, 1.30 mmol) in 1,2-dimethoxyethane (DME) (12 mL), and the mixture was stirred at 0 °C for 1 h and then at rt for 15 h.

Saturated aqueous $NaHCO_3$ solution was added to the mixture and the aqueous solution was extracted with diethyl ether (30 mL × 3). The combined organic solution was washed with saturated aqueous NaCl solution, dried over anhydrous $MgSO_4$, and the solution was concentrated under reduced pressure. The residue was purified by silica gel column chromatography [hexane : ethyl acetate (3 : 1)] to afford **5** (2.16 g, 85%) as a light yellow solid.

^{1}H NMR (300 MHz, $CDCl_3$) δ 10.33 (s, 1H), 7.41 (t, *J* = 8.3 Hz, 1H), 6.63 (dd, *J* = 8.5, 0.8 Hz, 1H), 6.44 (dd, *J* = 8.2, 0.8 Hz, 1H), 1.75 (s, 6H); ^{13}C NMR (176 MHz, $CDCl_3$) δ 165.49 (s), 161.44 (s), 155.60 (s), 137.93 (s), 110.82 (s), 107.25 (s), 107.13 (s), 99.38 (s), 25.66 (s); MS (ESI) *m/z*: 193 $[M+H]^-$; HRMS (ESI) *m/z*: Calcd. for $[M+H]^+$ $C_{10}H_{10}O_4$: 194.06; found: 194.05797.

Synthesis of 2-hydroxy-6-(alkoxy)benzoic acid (1a-f).

5-Hydroxy-2,2-dimethyl-4H-benzo[d][1,3]dioxin-4-one (**5**) (100 mg, 0.52 mmol) and K_2CO_3 (287 mg, 2.08 mmol) were suspended in 2.5 mL DMF under argon atmosphere. Suspension of 1-bromoalkane (0.78 mmol) in 2.7 mL DMF was added to the above suspension and stirred at 60 °C for 24 h. The above mixture was diluted with demineralized water (30 mL) and extracted with ethyl acetate (30 mL × 3). The combined organic layers were washed with demineralized water (20 mL × 2), 0.1 M aqueous HCl (20 mL) and saturated aqueous NaCl solution, dried over anhydrous $MgSO_4$, and the solution was concentrated under reduced pressure to afford **6a-f** as a white solid. The residue was dissolved in THF (10 mL) and 5 M aqueous KOH solution (1 mL) was added to the solution. The reaction mixture was stirred at 60 °C for 24 h. The mixture was acidified with 1 N aqueous HCl. The above mixture was extracted with ethyl acetate (40 mL × 2). The combined organic layers were washed with demineralized water (50 mL), brine (30 mL × 2), dried over anhydrous

$MgSO_4$, and the solution was concentrated under reduced pressure. The residue was purified by silica gel column chromatography [hexane : ethyl acetate (4 : 1)] to afford **1a-f** as a light yellow solid.

Synthesis of 2-hydroxy-6-(nonyloxy)benzoic acid (1a).

5-Hydroxy-2,2-dimethyl-4H-benzo[d][1,3]dioxin-4-one (**5**) (100 mg, 0.52 mmol) and K_2CO_3 (287 mg, 2.08 mmol) was suspended in 2.5 mL DMF under argon atmosphere. Suspension of 1-bromononane (0.78 mmol) in 2.7 mL DMF was added to the above suspension and stirred at 60 °C for 24 h. The above mixture was diluted with demineralized water (30 mL) and extracted with ethyl acetate (30 mL × 3). The combined organic layers were washed with demineralized water (20 mL × 2), 0.1 M aqueous HCl (20 mL) and saturated aqueous NaCl solution, dried over anhydrous $MgSO_4$, and the solution was concentrated under reduced pressure. The residue was dissolved in THF (10 mL) and 5 M aqueous KOH solution (1 mL) was added to the solution. The reaction mixture was stirred at 60 °C for 24 h. The mixture was acidified with 1 N aqueous HCl. The above mixture was extracted with ethyl acetate (40 mL × 2). The combined organic layers were washed with demineralized water (50 mL), brine (30 mL × 2), dried over anhydrous $MgSO_4$, and the solution was concentrated under reduced pressure. The residue was purified by silica gel column chromatography [hexane : ethyl acetate (4 : 1)] to afford **1a** (72 mg, 50%) as a light yellow solid.

^1H NMR (300 MHz, $CDCl_3$) δ 12.15 (s, 1H), 11.54 (s, 1H), 7.38 (t, J = 8.4 Hz, 1H), 6.69 (d, J = 8.5 Hz, 1H), 6.47 (d, J = 8.3 Hz, 1H), 4.23 (t, J = 6.6 Hz, 2H), 1.96 – 1.85 (m, 2H), 1.52 – 1.42 (m, 2H), 1.37 – 1.23 (m, 10H), 0.88 (t, J = 5.7 Hz, 3H); ^{13}C NMR (176 MHz, $CDCl_3$) δ 170.98 (s), 164.29 (s), 158.07 (s), 135.54 (s), 112.17 (s), 102.18 (s), 101.77 (s), 70.79 (s), 31.81 (s), 29.37 (s), 29.18 (s), 29.15 (s), 28.85 (s), 25.84 (s), 22.64 (s), 14.08 (s); MS (ESI) *m/z*: 279 $[M+H]^-$; HRMS (ESI) *m/z*: Calcd. for $[M+H]^+$ $C_{16}H_{24}O_4$: 280.17; found: 280.1674.

Synthesis of 2-(decyloxy)-6-hydroxybenzoic acid (1b).

5-Hydroxy-2,2-dimethyl-4H-benzo[d][1,3]dioxin-4-one (**5**) (100 mg, 0.52 mmol) and K_2CO_3 (287 mg, 2.08 mmol) was suspended in 2.5 mL DMF under argon atmosphere. Suspension of 1-bromodecane (0.78 mmol) in 2.7 mL DMF was added to the above suspension and stirred at 60 °C for 24 h. The above mixture was diluted with demineralized water (30 mL) and extracted with ethyl acetate (30 mL × 3). The combined organic layers were washed with demineralized water (20 mL × 2), 0.1 M aqueous HCl (20 mL) and saturated aqueous NaCl solution, dried over anhydrous $MgSO_4$, and the solution was concentrated under reduced pressure. The residue was dissolved in THF (10 mL) and 5 M aqueous KOH solution (1 mL) was added to the solution. The reaction mixture was stirred at 60 °C for 24 h. The mixture was acidified with 1 N aqueous HCl. The above mixture was extracted with ethyl acetate (40 mL × 2). The combined organic layers were washed with demineralized water (50 mL), brine (30 mL × 2), dried over anhydrous $MgSO_4$, and the solution was concentrated under reduced pressure. The residue was purified by silica gel column chromatography [hexane : ethyl acetate (4 : 1)] to afford **1b** (111 mg, 73%) as a light yellow solid.

^1H NMR (300 MHz, $CDCl_3$) δ 12.14 (s, 1H), 7.37 (t, J = 8.4 Hz, 1H), 6.69 (d, J = 8.5 Hz, 1H), 6.46 (d, J = 8.3 Hz, 1H), 4.21 (t, J = 6.6 Hz, 2H), 1.95 – 1.84 (m, 2H), 1.51 – 1.41 (m, 2H), 1.33 – 1.23 (m, 12H), 0.87 (t, J = 6.6 Hz, 3H); ^{13}C NMR (176 MHz,

$CDCl_3$) δ 170.95 (s), 164.28 (s), 158.06 (s), 135.52 (s), 112.17 (s), 102.16 (s), 101.76 (s), 70.78 (s), 31.84 (s), 29.44 (s), 29.39 (s), 29.24 (s), 29.16 (s), 28.84 (s), 25.83 (s), 22.65 (s), 14.07 (s); MS (ESI) *m/z*: 293 $[M+H]^-$; HRMS (ESI) *m/z*: Calcd. for $[M+H]^+$ $C_{17}H_{26}O_4$: 294.18; found: 294.1829.

Synthesis of 2-hydroxy-6-(undecyloxy)benzoic acid (1c).

5-Hydroxy-2,2-dimethyl-4H-benzo[d][1,3]dioxin-4-one (**5**) (100 mg, 0.52 mmol) and K_2CO_3 (287 mg, 2.08 mmol) was suspended in 2.5 mL DMF under argon atmosphere. Suspension of 1-bromoundecane (0.78 mmol) in 2.7 mL DMF was added to the above suspension and stirred at 60 °C for 24 h. The above mixture was diluted with demineralized water (30 mL) and extracted with ethyl acetate (30 mL × 3). The combined organic layers were washed with demineralized water (20 mL × 2), 0.1 M aqueous HCl (20 mL) and saturated aqueous NaCl solution, dried over anhydrous $MgSO_4$, and the solution was concentrated under reduced pressure. The residue was dissolved in THF (10 mL) and 5 M aqueous KOH solution (1 mL) was added to the solution. The reaction mixture was stirred at 60 °C for 24 h. The mixture was acidified with 1 N aqueous HCl. The above mixture was extracted with ethyl acetate (40 mL × 2). The combined organic layers were washed with demineralized water (50 mL), brine (30 mL × 2), dried over anhydrous $MgSO_4$, and the solution was concentrated under reduced pressure. The residue was purified by silica gel column chromatography [hexane : ethyl acetate (4 : 1)] to afford **1c** (146 mg, 92%) as a light yellow solid.

^1H NMR (300 MHz, $CDCl_3$) δ 12.15 (s, 1H), 11.54 (s, 1H), 7.37 (t, J = 7.9 Hz, 1H), 6.68 (d, J = 8.5 Hz, 1H), 6.47 (d, J = 8.4 Hz, 1H), 4.22 (t, J = 6.6 Hz, 2H), 1.96 – 1.85 (m, 2H), 1.55 – 1.42 (m, 2H), 1.36 – 1.24 (m, 14H), 0.88 (t, J = 7.0 Hz, 3H); ^{13}C NMR (176 MHz, $CDCl_3$) δ 170.98 (s), 164.28 (s), 158.08 (s), 135.55 (s), 112.16 (s), 102.19 (s), 101.75 (s), 70.80 (s), 31.90 (s), 29.56 (s), 29.51 (s), 29.41 (s), 29.30 (s), 29.18 (s), 28.86 (s), 25.84 (s), 22.68 (s), 14.10 (s); MS (ESI) *m/z*: 307 $[M+H]^-$; HRMS (ESI) *m/z*: Calcd. for $[M+H]^+$ $C_{18}H_{28}O_4$: 308.20; found: 308.1986.

Synthesis of 2-(dodecyloxy)-6-hydroxybenzoic acid (1d).

5-Hydroxy-2,2-dimethyl-4H-benzo[d][1,3]dioxin-4-one (**5**) (100 mg, 0.52 mmol) and K_2CO_3 (287 mg, 2.08 mmol) was suspended in 2.5 mL DMF under argon atmosphere. Suspension of 1-bromododecane (0.78 mmol) in 2.7 mL DMF was added to the above suspension and stirred at 60 °C for 24 h. The above mixture was diluted with demineralized water (30 mL) and extracted with ethyl acetate (30 mL × 3). The combined organic layers were washed with demineralized water (20 mL × 2), 0.1 M aqueous HCl (20 mL) and saturated aqueous NaCl solution, dried over anhydrous $MgSO_4$, and the solution was concentrated under reduced pressure. The residue was dissolved in THF (10 mL) and 5 M aqueous KOH solution (1 mL) was added to the solution. The reaction mixture was stirred at 60 °C for 24 h. The mixture was acidified with 1 N aqueous HCl. The above mixture was extracted with ethyl acetate (40 mL × 2). The combined organic layers were washed with demineralized water (50 mL), brine (30 mL × 2), dried over anhydrous $MgSO_4$, and the solution was concentrated under reduced pressure. The residue was purified by silica gel column chromatography [hexane : ethyl acetate (4 : 1)] to afford **1d** (121 mg, 73%) as a light yellow solid.

^1H NMR (300 MHz, $CDCl_3$) δ 12.13 (s, 1H), 7.36 (t, J = 8.4 Hz, 1H), 6.68 (d, J = 8.5 Hz, 1H), 6.45 (d, J = 8.3 Hz, 1H), 4.20 (t,

J = 6.6 Hz, 2H), 1.95 – 1.82 (m, 2H), 1.51 – 1.40 (m, 2H), 1.31 – 1.20 (m, 16H), 0.85 (t, J = 4.9 Hz, 3H); ^{13}C NMR (176 MHz, $CDCl_3$) δ 170.96 (s), 164.26 (s), 158.05 (s), 135.53 (s), 112.13 (s), 102.17 (s), 101.72 (s), 70.78 (s), 31.89 (s), 29.59 (s), 29.49 (s), 29.40 (s), 29.32 (s), 29.16 (s), 28.83 (s), 25.82 (s), 22.67 (s), 14.08 (s); MS (ESI) *m/z*: 321 $[M+H]^-$; HRMS (ESI) *m/z*: Calcd. for $[M+H]^+$ $C_{19}H_{30}O_4$: 322.17; found: 322.2140.

Synthesis of 2-hydroxy-6-(tridecyloxy)benzoic acid (1e).

5-Hydroxy-2,2-dimethyl-4H-benzo[d][1,3]dioxin-4-one (**5**) (100 mg, 0.52 mmol) and K_2CO_3 (287 mg, 2.08 mmol) was suspended in 2.5 mL DMF under argon atmosphere. Suspension of 1-bromotridecane (0.78 mmol) in 2.7 mL DMF was added to the above suspension and stirred at 60 °C for 24 h. The above mixture was diluted with demineralized water (30 mL) and extracted with ethyl acetate (30 mL × 3). The combined organic layers were washed with demineralized water (20 mL × 2), 0.1 M aqueous HCl (20 mL) and saturated aqueous NaCl solution, dried over anhydrous $MgSO_4$, and the solution was concentrated under reduced pressure. The residue was dissolved in THF (10 mL) and 5 M aqueous KOH solution (1 mL) was added to the solution. The reaction mixture was stirred at 60 °C for 24 h. The mixture was acidified with 1 N aqueous HCl. The above mixture was extracted with ethyl acetate (40 mL × 2). The combined organic layers were washed with demineralized water (50 mL), brine (30 mL × 2), dried over anhydrous $MgSO_4$, and the solution was concentrated under reduced pressure. The residue was purified by silica gel column chromatography [hexane : ethyl acetate (4 : 1)] to afford **1e** (170 mg, 98%) as a light yellow solid.

^{1}H NMR (300 MHz, $CDCl_3$) δ 12.15 (s, 1H), 11.59 (s, 1H), 7.37 (t, J = 8.4 Hz, 1H), 6.68 (d, J = 8.5 Hz, 1H), 6.47 (d, J = 8.3 Hz, 1H), 4.22 (t, J = 6.6 Hz, 2H), 1.97 – 1.84 (m, 2H), 1.54 – 1.42 (m, 2H), 1.37 – 1.23 (m, 18H), 0.88 (t, J = 6.6 Hz, 3H); ^{13}C NMR (176 MHz, $CDCl_3$) δ 170.95 (s), 164.27 (s), 158.05 (s), 135.53 (s), 112.16 (s), 102.16 (s), 101.74 (s), 70.78 (s), 31.91 (s), 29.63 (s), 29.62 (s), 29.59 (s), 29.49 (s), 29.40 (s), 29.33 (s), 29.16 (s), 28.84 (s), 25.83 (s), 22.68 (s), 14.09 (s); MS (ESI) *m/z*: 335 $[M+H]^-$; HRMS (ESI) *m/z*: Calcd. for $[M+H]^+$ $C_{20}H_{32}O_4$: 336.23; found: 336.2300.

Synthesis of 2-hydroxy-6-(pentadecyloxy)benzoic acid (1f).

5-Hydroxy-2,2-dimethyl-4H-benzo[d][1,3]dioxin-4-one (**5**) **(**100 mg, 0.52 mmol) and K_2CO_3 (287 mg, 2.08 mmol) was suspended in 2.5 mL DMF under argon atmosphere. Suspension of 1-bromopentadecane (0.78 mmol) in 2.7 mL DMF was added to the above suspension and stirred at 60 °C for 24 h. The above mixture was diluted with demineralized water (30 mL) and extracted with ethyl acetate (30 mL × 3). The combined organic layers were washed with demineralized water (20 mL × 2), 0.1 M aqueous HCl (20 mL) and saturated aqueous NaCl solution, dried over anhydrous $MgSO_4$, and the solution was concentrated under reduced pressure. The residue was dissolved in THF (10 mL) and 5 M aqueous KOH solution (1 mL) was added to the solution. The reaction mixture was stirred at 60 °C for 24 h. The mixture was acidified with 1 N aqueous HCl. The above mixture was extracted with ethyl acetate (40 mL × 2). The combined organic layers were washed with demineralized water (50 mL), brine (30 mL × 2), dried over anhydrous $MgSO_4$, and the solution was concentrated under reduced pressure. The residue was purified by silica gel column chromatography [hexane : ethyl acetate (4 : 1)] to afford **1f** (172 mg, 92%) as a light yellow solid.

^1H NMR (300 MHz, $CDCl_3$) δ 12.17 (s, 1H), 11.61 (s, 1H), 7.38 (t, J = 8.4 Hz, 1H), 6.71 (d, J = 8.5 Hz, 1H), 6.47 (d, J = 8.3 Hz, 1H), 4.23 (t, J = 6.6 Hz, 2H), 1.97 – 1.85 (m, 2H), 1.53 – 1.42 (m, 2H), 1.35 – 1.20 (m, 22H), 0.88 (t, J = 5.8 Hz, 3H); ^{13}C NMR (176 MHz, $CDCl_3$) δ 170.96 (s), 164.28 (s), 158.06 (s), 135.53 (s), 112.17 (s), 102.15 (s), 101.75 (s), 70.78 (s), 31.92 (s), 29.68 (s), 29.67 (s), 29.65 (s), 29.64 (s), 29.60 (s), 29.50 (s), 29.40 (s), 29.35 (s), 29.17 (s), 28.84 (s), 25.83 (s), 22.69 (s), 14.10 (s); MS (ESI) *m/z*: 363 $[M+H]^-$; HRMS (ESI) *m/z*: Calcd. for $[M+H]^+$ $C_{22}H_{36}O_4$: 364.26; found: 364.2615.

Synthesis of 7-hydroxy-2,2-dimethyl-4H-benzo[d][1,3]dioxin-4-one (8).

Under argon atmosphere, acetone (1.21 mL, 16.23 mmol) and trifluoroacetic anhydride (6 mL, 42.83 mmol) were added to a solution of 2,4-dihydroxybenzoic acid (**7**) (1.0 g, 6.49 mmol) in 10 mL trifluoroacetic acid at 0 °C and stirred at 60 °C for 24 h. The above mixture was added to saturated aqueous $NaHCO_3$ (50 mL) and extracted with ethyl acetate (30 mL × 3). The combined organic layers were washed with demineralized water (50 mL) and saturated aqueous NaCl solution, dried over anhydrous $MgSO_4$, and the solution was concentrated under reduced pressure. The residue was purified by silica gel column chromatography [hexane : ethyl acetate (4 : 1)] to afford **8** (291 mg, 23%) as a white solid.

^1H NMR (300 MHz, $CDCl_3$) δ 7.72 (d, *J* = 8.6 Hz, 1H), 6.57 (dd, *J* = 8.6, 2.0 Hz, 1H), 6.34 (d, *J* = 2.0 Hz, 1H), 1.68 (s, 6H); ^{13}C NMR (176 MHz, $CDCl_3$) δ 162.79 (s), 161.03 (s), 158.04 (s), 131.79 (s), 111.00 (s), 106.60 (s), 106.41 (s), 103.24 (s), 25.82 (s); MS (ESI) *m/z*: 193 $[M+H]^-$; HRMS (ESI) *m/z*: Calcd. for $[M+H]^+$ $C_{10}H_{10}O_4$: 194.06; found: 194.0580.

Synthesis of 2-hydroxy-4-(tridecyloxy)benzoic acid (2) .

7-Hydroxy-2,2-dimethyl-4H-benzo[d][1,3]dioxin-4-one **(8)** (80 mg, 0.41 mmol) and K_2CO_3 (202 mg, 0.62 mmol) was suspended in 2 mL DMF under argon atmosphere. Suspension of 1-bromotridecane (158 μL, 0.62 mmol) in 2 mL DMF was added to the above suspension and stirred at 50 °C for 5.5 h. The above mixture was diluted with demineralized water (30 mL) and extracted with ethyl acetate (30 mL × 3). The combined organic layers were washed with demineralized water (20 mL × 2), 0.1 M aqueous HCl (20 mL) and saturated aqueous NaCl solution, dried over anhydrous $MgSO_4$, and the solution was concentrated under reduced pressure. The residue was dissolved in THF (10 mL) and 5 M aqueous KOH solution (1.2 mL) was added to the solution. The reaction mixture was stirred at 60 °C for 6 h. The mixture was acidified with 1 N aqueous HCl. The above mixture was extracted with ethyl acetate (40 mL × 2). The combined organic layers were washed with demineralized water (50 mL), brine (30 mL × 2), dried over anhydrous $MgSO_4$, and the solution was concentrated under reduced pressure. The residue was purified by silica gel column chromatography [hexane : ethyl acetate (4 : 1)] to afford **2** (102 mg, 74%) as a light yellow solid.

^1H NMR (300 MHz, $CDCl_3$) δ 10.60 (s, 1H), 7.81 (d, *J* = 8.7 Hz, 1H), 6.48 (dd, 1H), 6.44 (d, *J* = 2.3 Hz, 1H), 3.99 (t, *J* = 6.5 Hz, 2H), 1.85 – 1.74 (m, 2H), 1.50 – 1.39 (m, 2H), 1.26 (s, 18H), 0.88 (t, *J* = 6.7 Hz, 3H); ^{13}C NMR (176 MHz, $CDCl_3$) δ 174.21 (s), 166.29 (s), 164.53 (s), 132.25 (s), 108.54 (s), 104.04 (s), 101.26 (s), 68.45 (s), 31.92 (s), 29.67 (s), 29.64 (s), 29.57 (s), 29.53 (s), 29.35 (s), 29.32 (s), 28.97 (s), 25.94 (s), 22.68 (s), 14.10 (s); MS (ESI) *m/z*: 335 $[M+H]^-$; HRMS (FAB) *m/z*: Calcd. for $[M+H]^+$ $C_{20}H_{32}O_4$: 336.23; found: 337.2378.

Synthesis of 6-hydroxy-2,2-dimethyl-4H-benzo[d][1,3]dioxin-4-one (11).

Under argon atmosphere, acetone (1.21 mL, 16.23 mmol) and trifluoroacetic anhydride (6 mL, 42.83 mmol) were added to a solution of 2,5-dihydroxybenzoic acid (**10**) (1.0 g, 6.49 mmol) in 10 mL trifluoroacetic acid at 0 °C and stirred at 60 °C for 24 h. The above mixture was added to saturated aqueous $NaHCO_3$ (50 mL) and extracted with ethyl acetate (30 mL × 3). The combined organic layers were washed with demineralized water (50 mL) and saturated aqueous NaCl solution, dried over anhydrous $MgSO_4$, and the solution was concentrated under reduced pressure. The residue was purified by silica gel column chromatography [hexane : ethyl acetate (4 : 1)] to afford **11** (252 mg, 20%) as a white solid.

^{1}H NMR (300 MHz, $CDCl_3$) δ 7.50 (d, *J* = 3.0 Hz, 1H), 7.12 (dd, *J* = 8.8, 3.1 Hz, 1H), 6.86 (d, *J* = 8.8 Hz, 1H), 6.40 (s, 1H), 1.71 (s, 6H); ^{13}C NMR (176 MHz, $CDCl_3$) δ 162.04 (s), 151.40 (s), 149.92 (s), 124.85 (s), 118.40 (s), 114.48 (s), 113.69 (s), 106.73 (s), 25.63 (s); MS (ESI) *m*/*z*: 193 $[M+H]^-$; HRMS (ESI) *m*/*z*: Calcd. for $[M+H]^+$ $C_{10}H_{10}O_4$: 194.06; found: 194.0576.

Synthesis of 2-hydroxy-5-(tridecyloxy)benzoic acid (3) .

6-Hydroxy-2,2-dimethyl-4H-benzo[d][1,3]dioxin-4-one **(11)** (80 mg, 0.41 mmol) and Cs_2CO_3 (202 mg, 0.62 mmol) was suspended in 2 mL DMF under argon atmosphere. Suspension of 1-bromotridecane (158 μL, 0.62 mmol) in 2 mL DMF was added to the above suspension and stirred at 50 °C for 18 h. The above mixture was diluted with demineralized water (30 mL) and extracted with ethyl acetate (30 mL × 3). The combined organic layers were washed with demineralized water (20 mL × 2), 0.1 M aqueous HCl (20 mL) and saturated aqueous NaCl solution, dried over anhydrous $MgSO_4$, and the solution was concentrated under reduced pressure. The residue was dissolved in THF (10 mL) and 5 M aqueous KOH solution (1 mL) was added to the solution. The reaction mixture was stirred at 60 °C for 6 h. The mixture was acidified with 1 N aqueous HCl. The above mixture was extracted with ethyl acetate (40 mL × 2). The combined organic layers were washed with demineralized water (50 mL), brine (30 mL × 2), dried over anhydrous $MgSO_4$, and the solution was concentrated under reduced pressure. The residue was purified by silica gel column chromatography [hexane : ethyl acetate (4 : 1)] to afford **3** (127 mg, 92%) as a light yellow solid.

^{1}H NMR (300 MHz, $CDCl_3$) δ 10.00 (s, 1H), 7.35 (d, *J* = 3.1 Hz, 1H), 7.15 (dd, *J* = 9.1, 3.1 Hz, 1H), 6.94 (d, *J* = 9.1 Hz, 1H), 3.93 (t, *J* = 6.5 Hz, 2H), 1.81 – 1.72 (m, 2H), 1.47 – 1.42 (m, 2H), 1.26 (s, 18H), 0.88 (t, *J* = 6.7 Hz, 3H); ^{13}C NMR (176 MHz, $CDCl_3$) δ 174.27 (s), 156.71 (s), 151.81 (s), 126.18 (s), 118.80 (s), 113.32 (s), 110.75 (s), 68.93 (s), 31.94 (s), 29.69 (s), 29.67 (s), 29.66 (s), 29.61 (s), 29.58 (s), 29.40 (s), 29.36 (s), 29.27 (s), 26.03 (s), 22.70 (s), 14.11 (s); MS (ESI) *m*/*z*: 335 $[M+H]^-$; HRMS (FAB) *m*/*z*: Calcd. for $[M+H]^+$ $C_{20}H_{32}O_4$: 336.23; found: 337.2383.

^{1}H-NMR spectrum of 5-hydroxy-2,2-dimethyl-4H-benzo[d][1,3]dioxin-4-one (5).

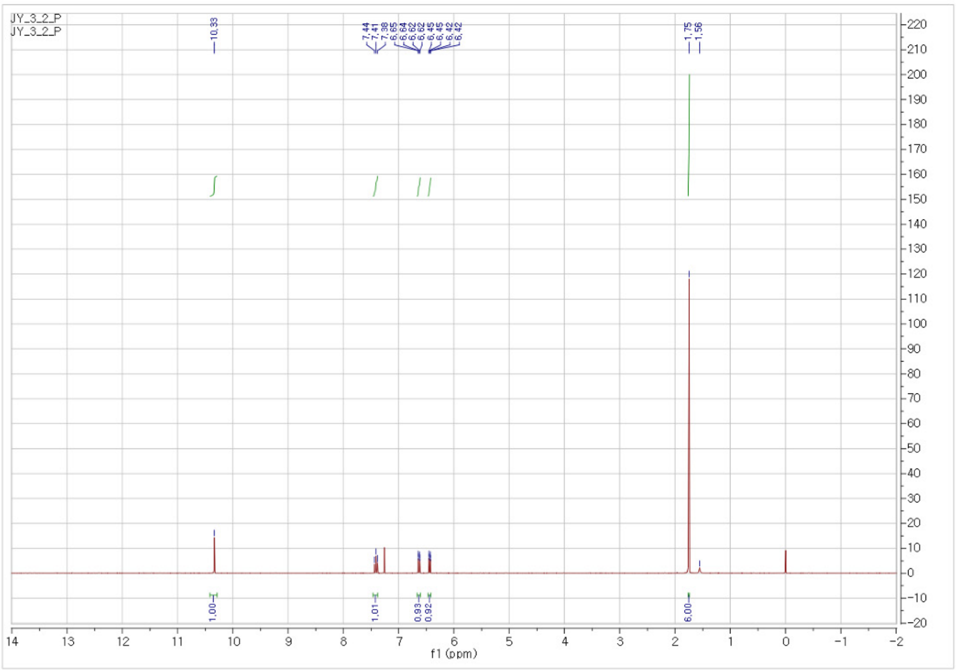

^{13}C-NMR spectrum of 5-hydroxy-2,2-dimethyl-4H-benzo[d][1,3]dioxin-4-one (5).

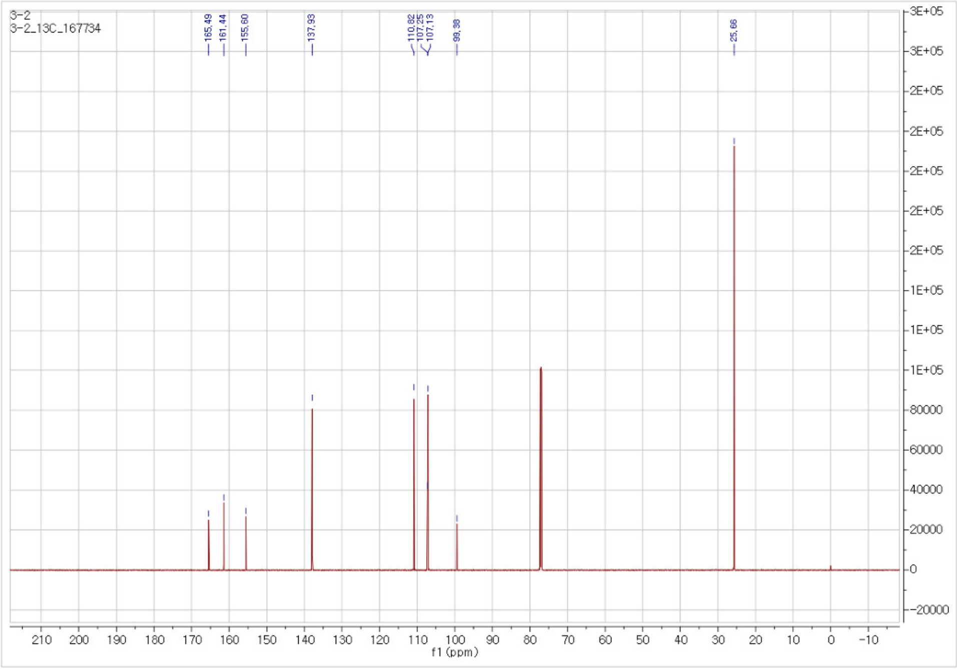

HRMS spectrum of 5-hydroxy-2,2-dimethyl-4H-benzo[d][1,3]dioxin-4-one (5).

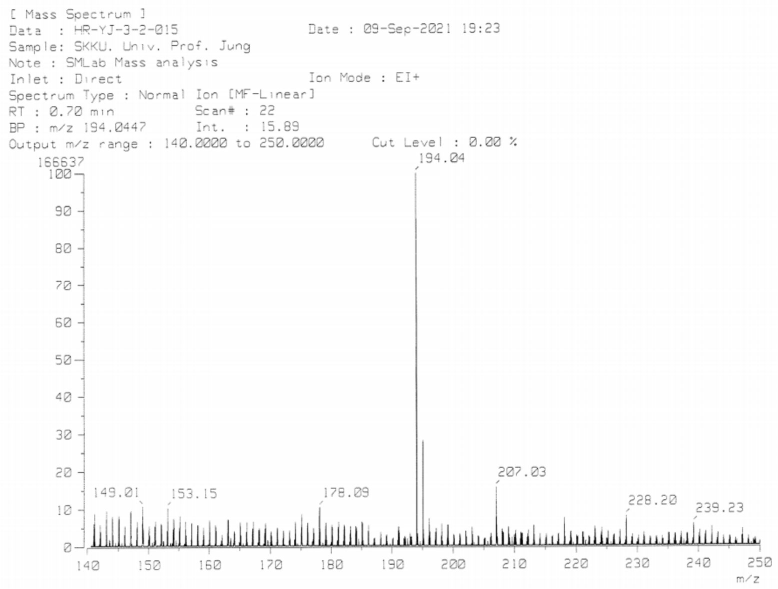

^{1}H-NMR spectrum of 2-hydroxy-6-(nonyloxy)benzoic acid (1a).

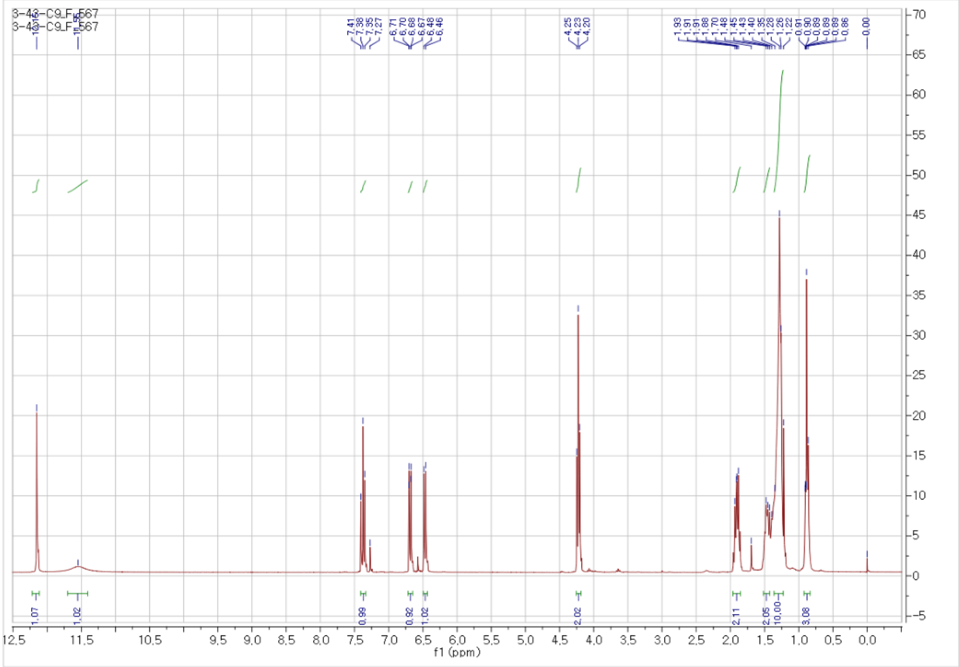

^{13}C-NMR spectrum of 2-hydroxy-6-(nonyloxy)benzoic acid (1a).

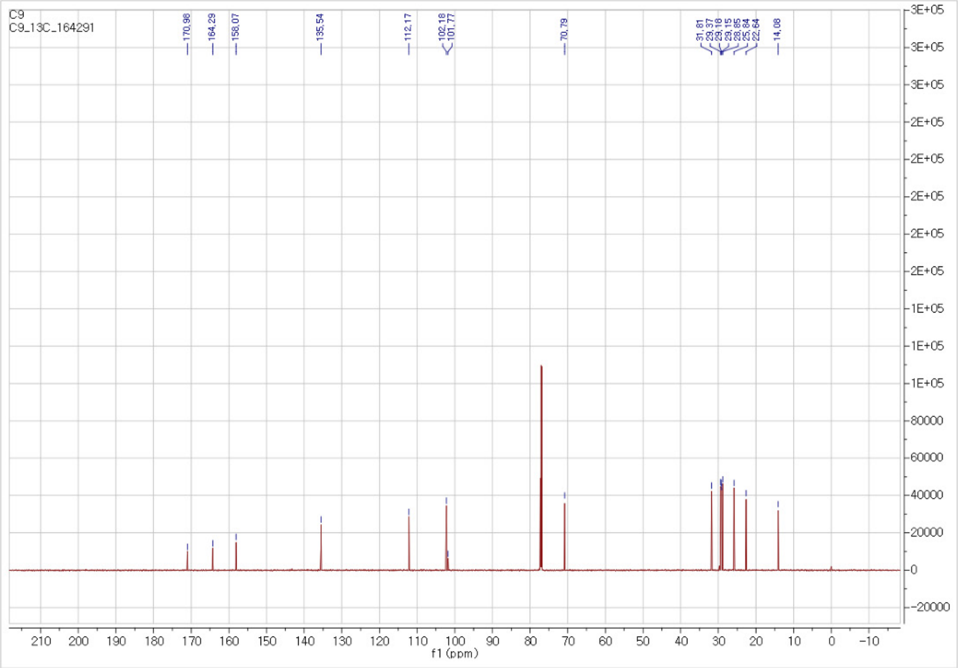

HRMS spectrum of 2-hydroxy-6-(nonyloxy)benzoic acid (1a).

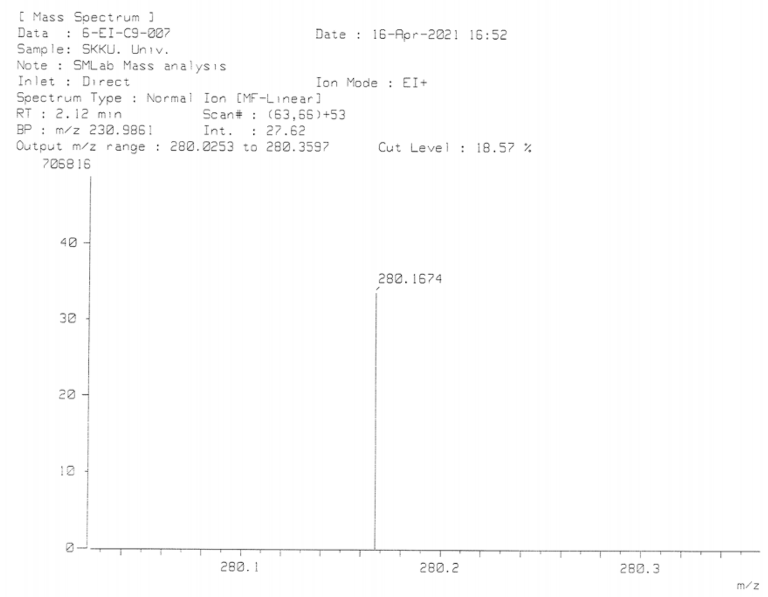

HPLC data of 2-hydroxy-6-(nonyloxy)benzoic acid (1a).

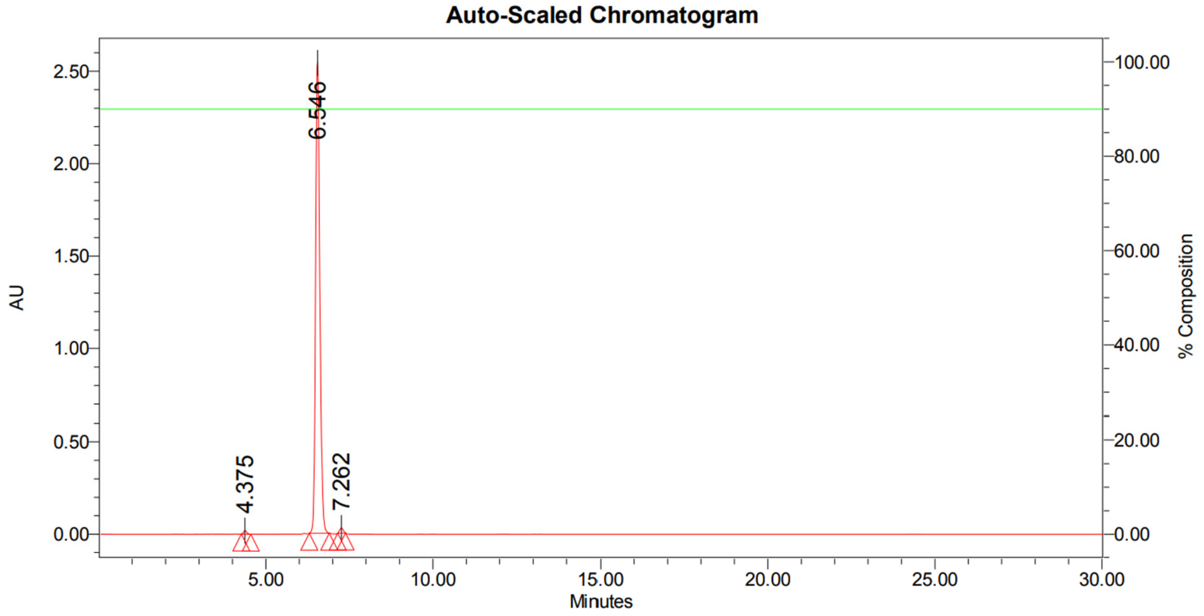

Processed Channel: PDA 254.0 nm

	Processed Channel	Retention Time (min)	Area	% Area	Height
1	PDA 254.0 nm	4.375	123398	0.54	19514
2	PDA 254.0 nm	6.546	22430413	98.56	2554779
3	PDA 254.0 nm	7.262	205320	0.90	27569

^{1}H-NMR spectrum of 2-(decyloxy)-6-hydroxybenzoic acid (1b).

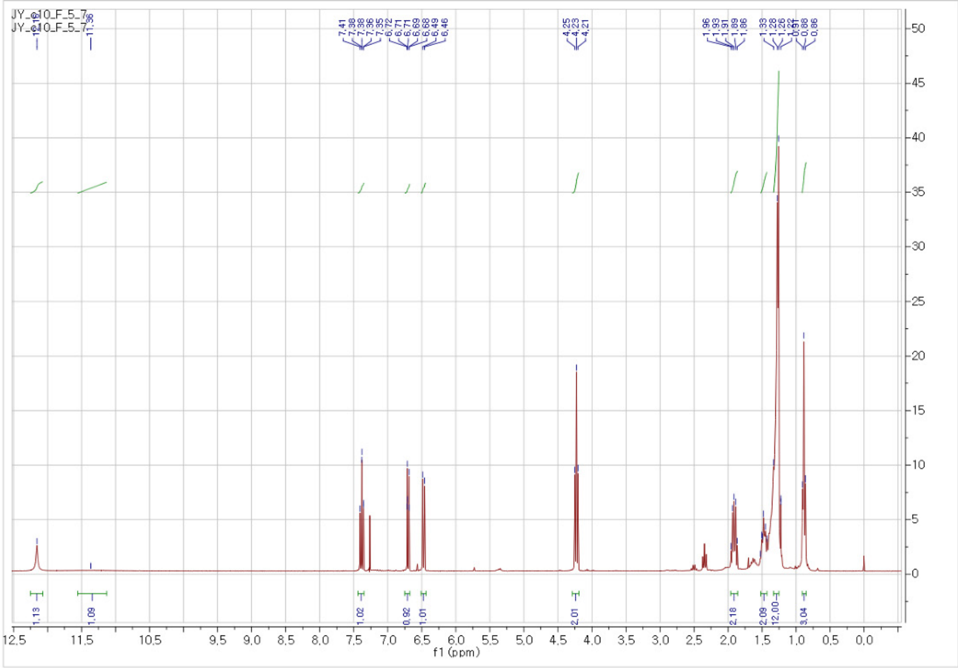

^{13}C-NMR spectrum of 2-(decyloxy)-6-hydroxybenzoic acid (1b).

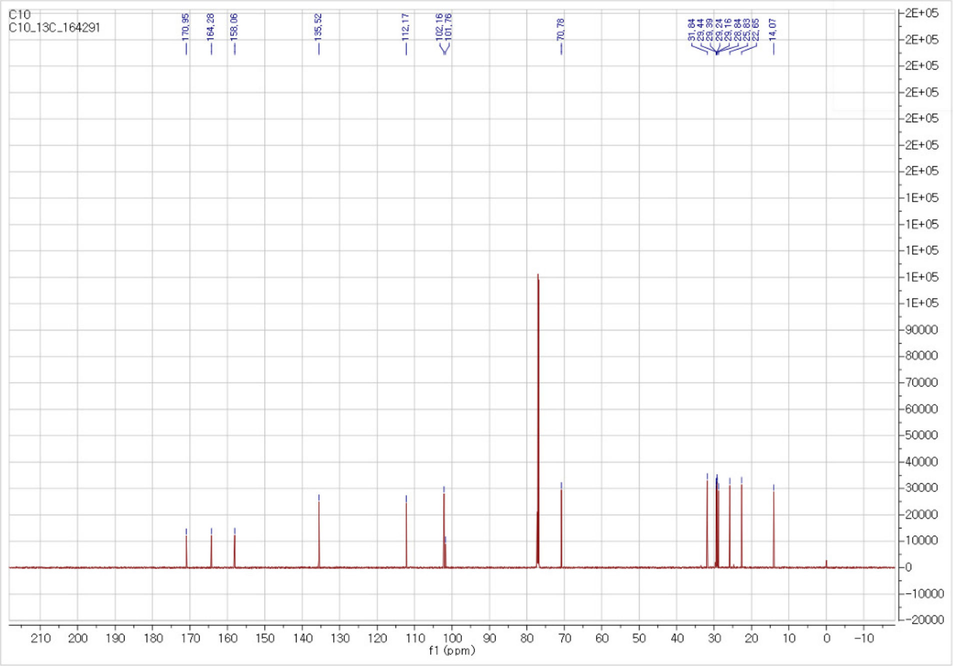

HRMS spectrum of 2-(decyloxy)-6-hydroxybenzoic acid (1b).

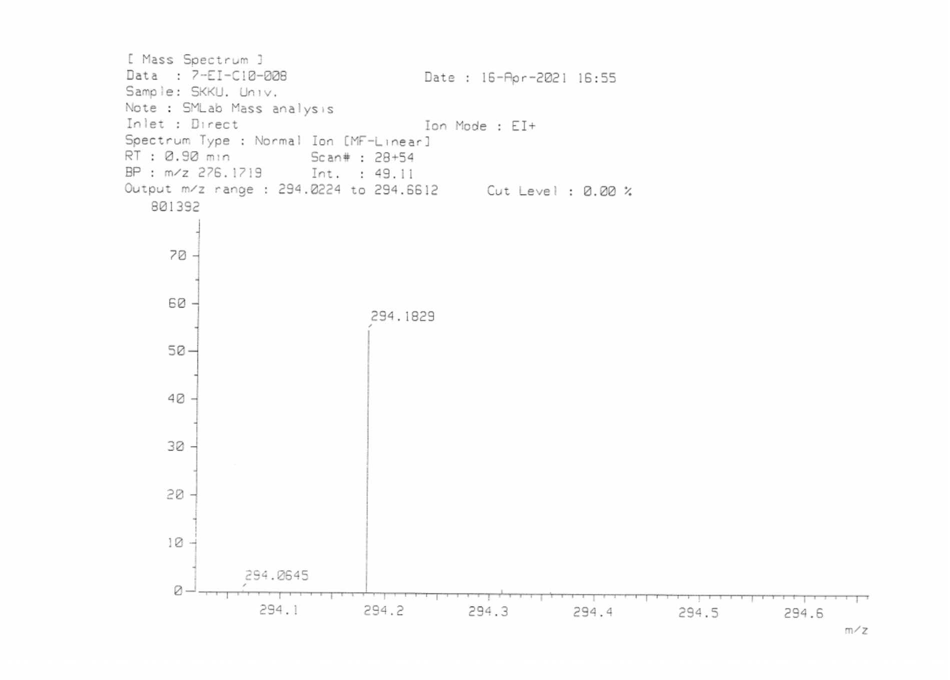

HPLC data of 2-(decyloxy)-6-hydroxybenzoic acid (1b).

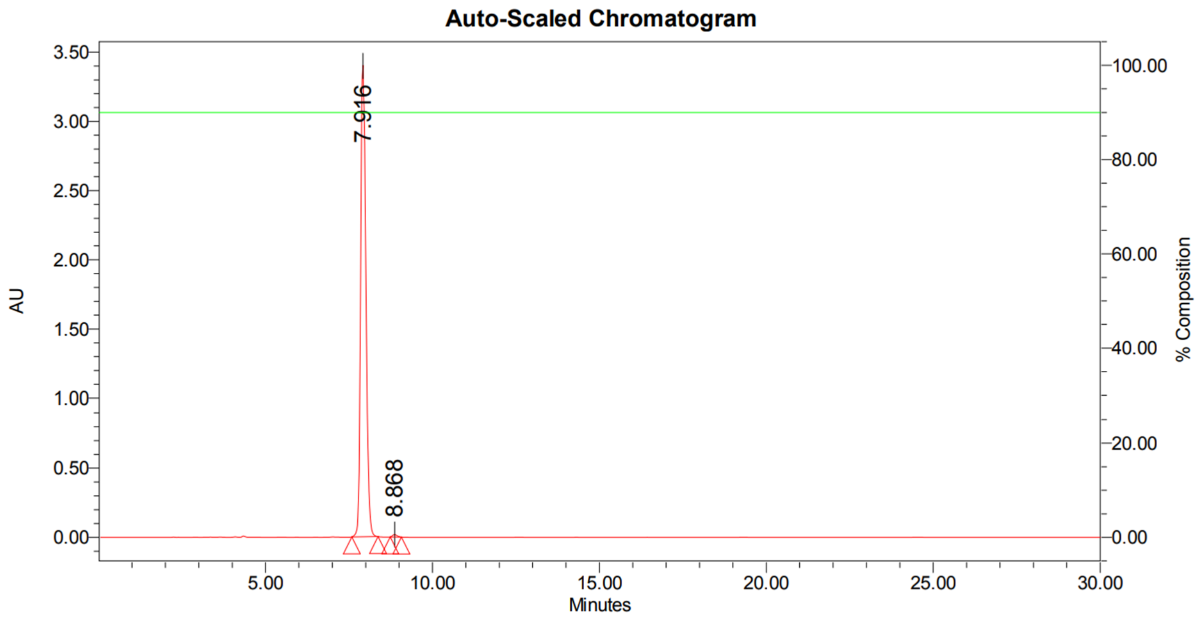

Processed Channel: PDA 254.0 nm

	Processed Channel	Retention Time (min)	Area	% Area	Height
1	PDA 254.0 nm	7.916	37176775	99.55	3370060
2	PDA 254.0 nm	8.868	166834	0.45	17423

^{1}H-NMR spectrum of 2-hydroxy-6-(undecyloxy)benzoic acid (1c).

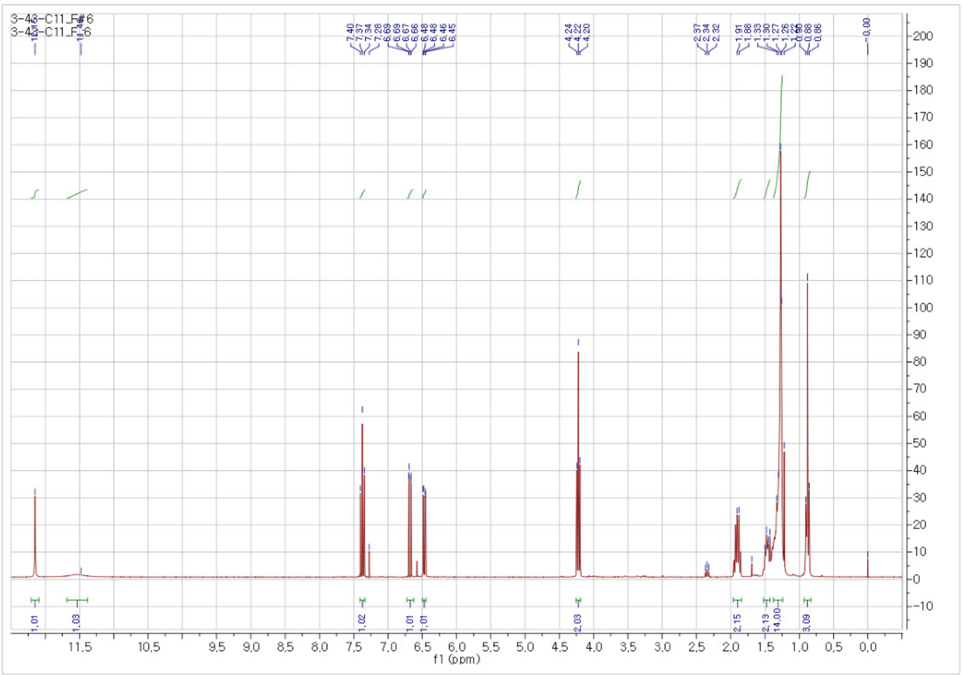

^{13}C-NMR spectrum of 2-hydroxy-6-(undecyloxy)benzoic acid (1c).

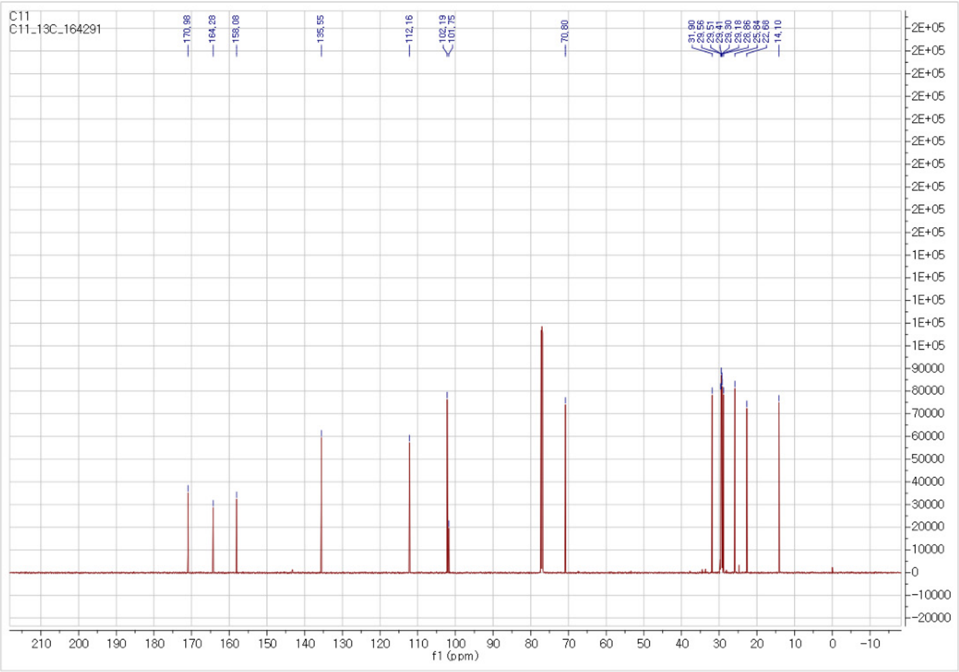

HRMS spectrum of 2-hydroxy-6-(undecyloxy)benzoic acid (1c).

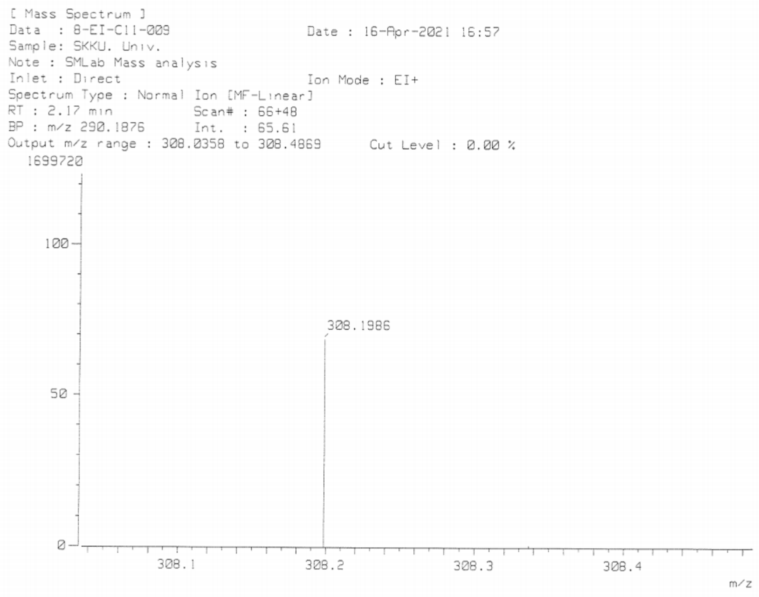

HPLC data of 2-hydroxy-6-(undecyloxy)benzoic acid (1c).

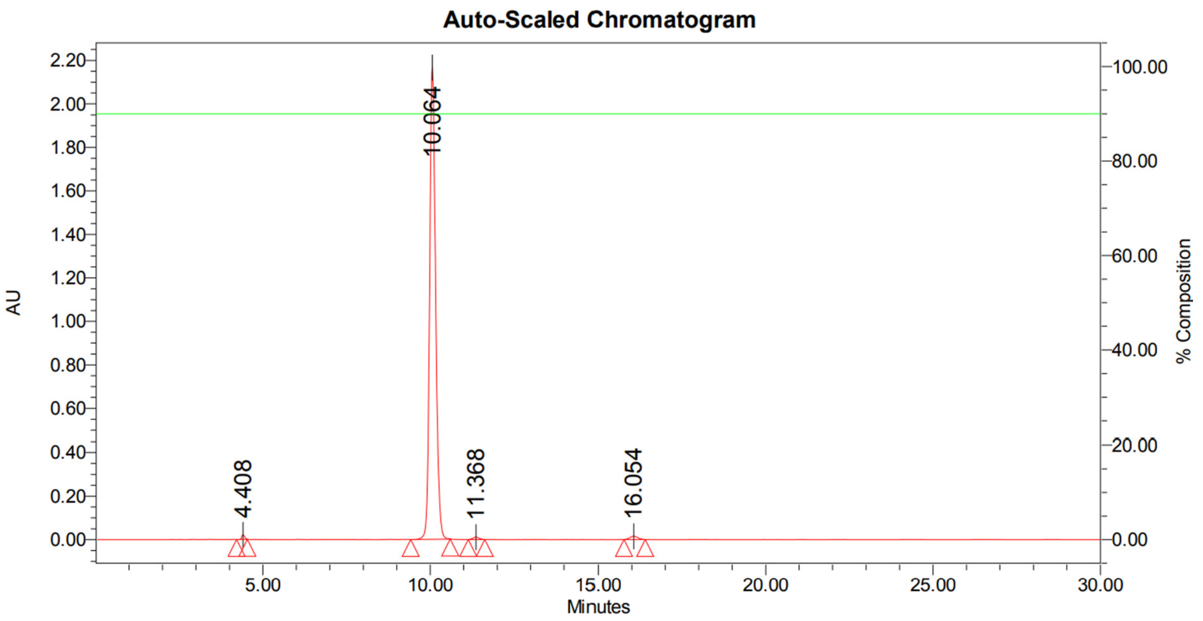

Processed Channel: PDA 254.0 nm

	Processed Channel	Retention Time (min)	Area	% Area	Height
1	PDA 254.0 nm	4.408	129409	0.47	20965
2	PDA 254.0 nm	10.064	26788537	98.10	2170053
3	PDA 254.0 nm	11.368	137887	0.50	10815
4	PDA 254.0 nm	16.054	252191	0.92	15012

^{1}H-NMR spectrum of 2-(dodecyloxy)-6-hydroxybenzoic acid (1d).

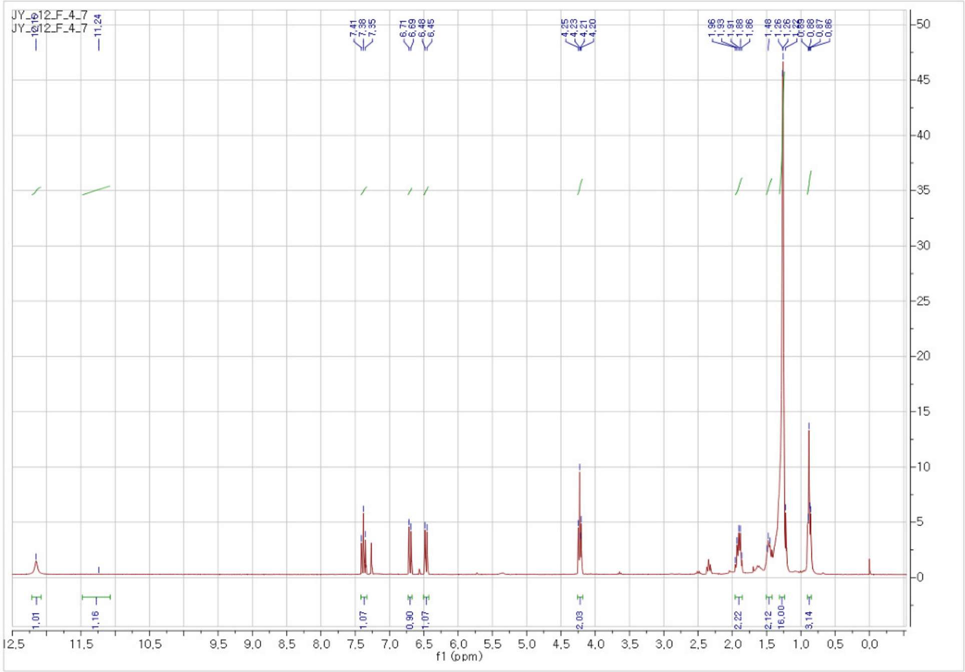

^{13}C-NMR spectrum of 2-(dodecyloxy)-6-hydroxybenzoic acid (1d).

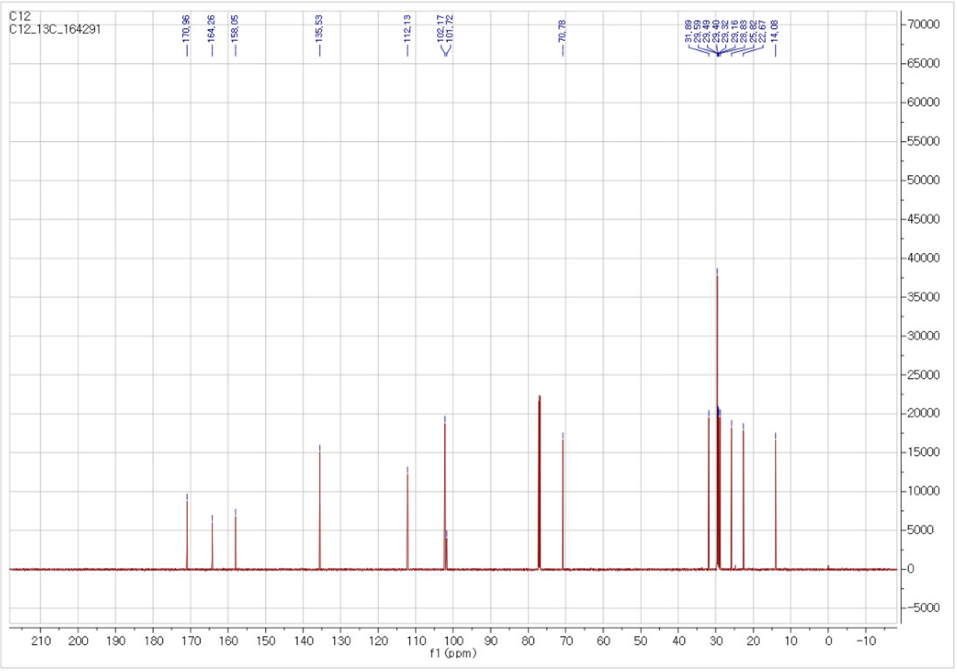

HRMS spectrum of 2-(dodecyloxy)-6-hydroxybenzoic acid (1d).

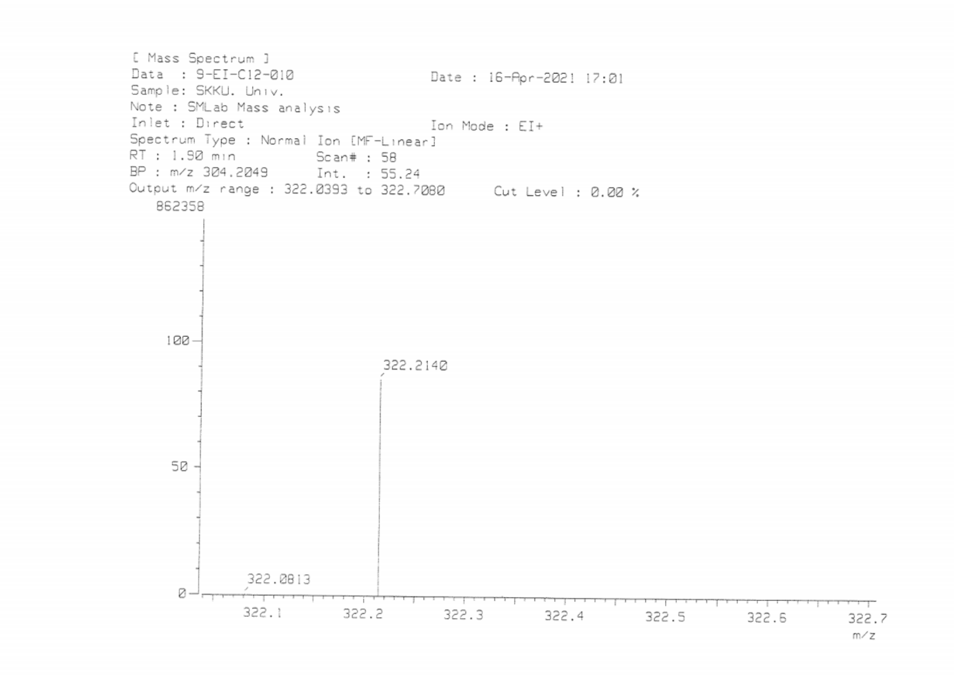

HPLC data of 2-(dodecyloxy)-6-hydroxybenzoic acid (1d).

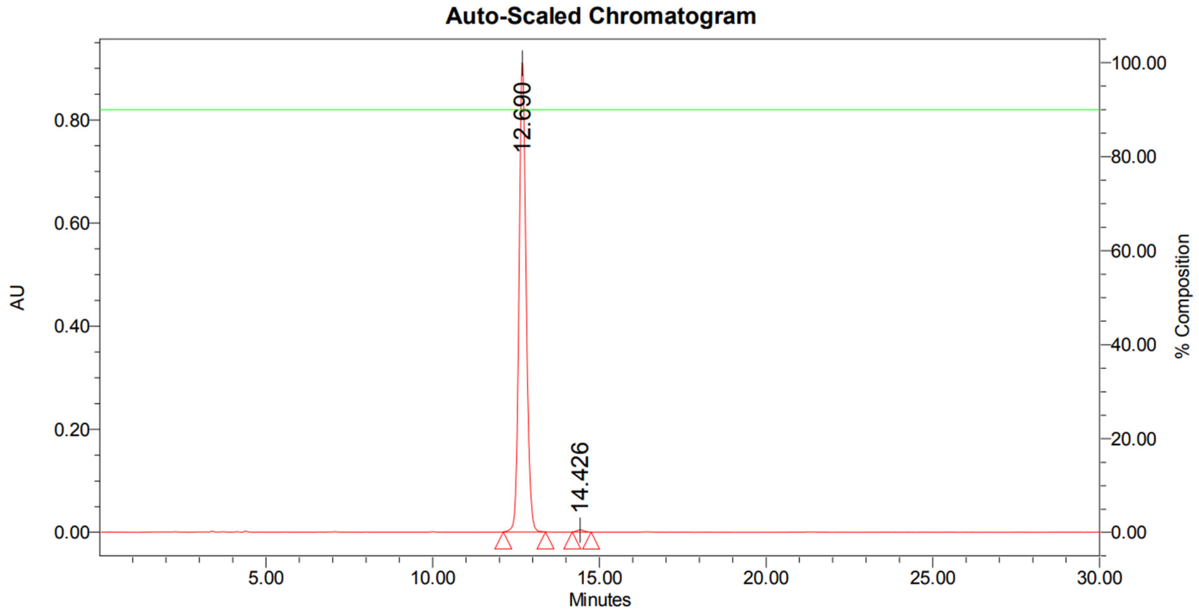

Processed Channel: PDA 254.0 nm

	Processed Channel	Retention Time (min)	Area	% Area	Height
1	PDA 254.0 nm	12.690	13517990	99.52	913001
2	PDA 254.0 nm	14.426	64981	0.48	4273

^{1}H-NMR spectrum of 2-hydroxy-6-(tridecyloxy)benzoic acid (1e).

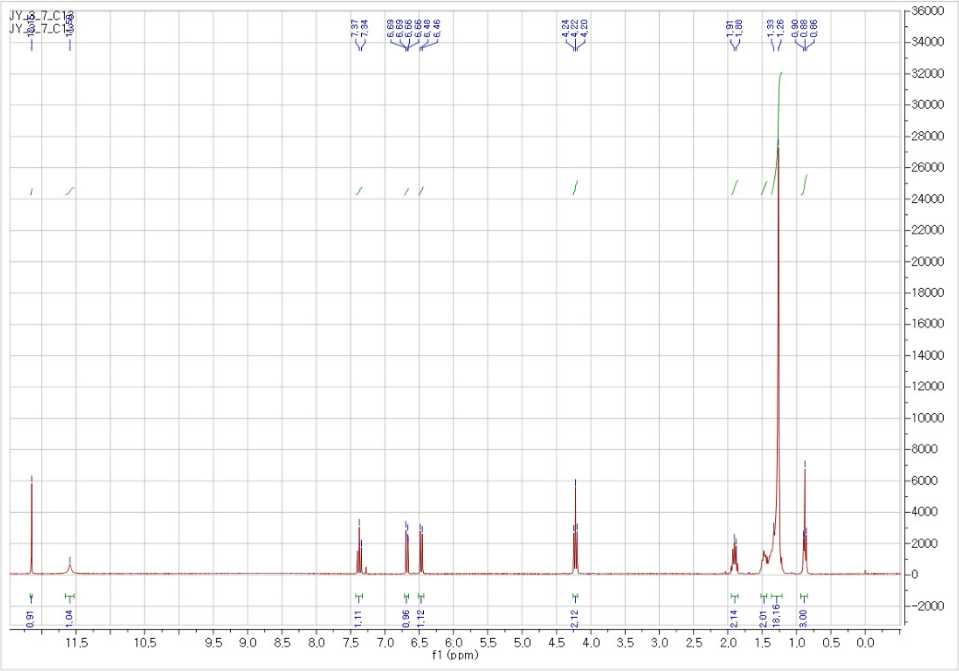

^{13}C-NMR spectrum of 2-hydroxy-6-(tridecyloxy)benzoic acid (1e).

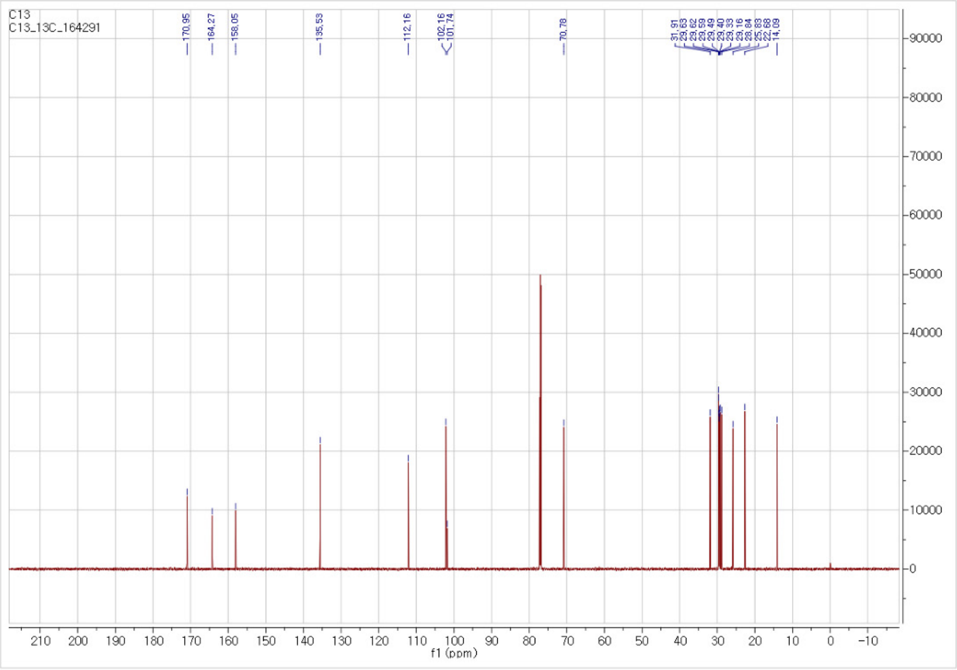

HRMS spectrum of 2-hydroxy-6-(tridecyloxy)benzoic acid (1e).

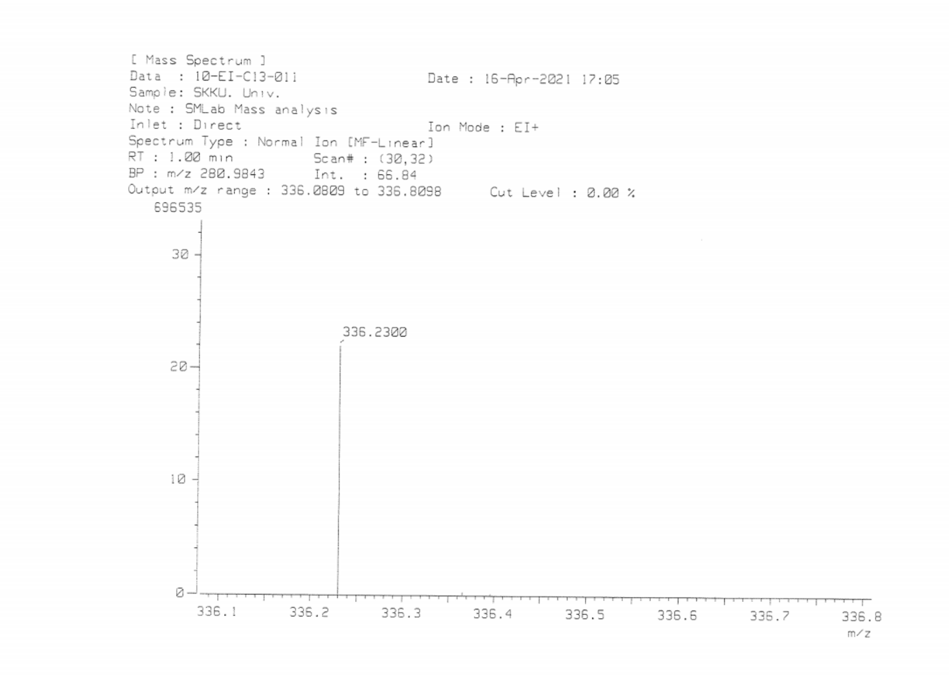

HPLC data of 2-hydroxy-6-(tridecyloxy)benzoic acid (1e).

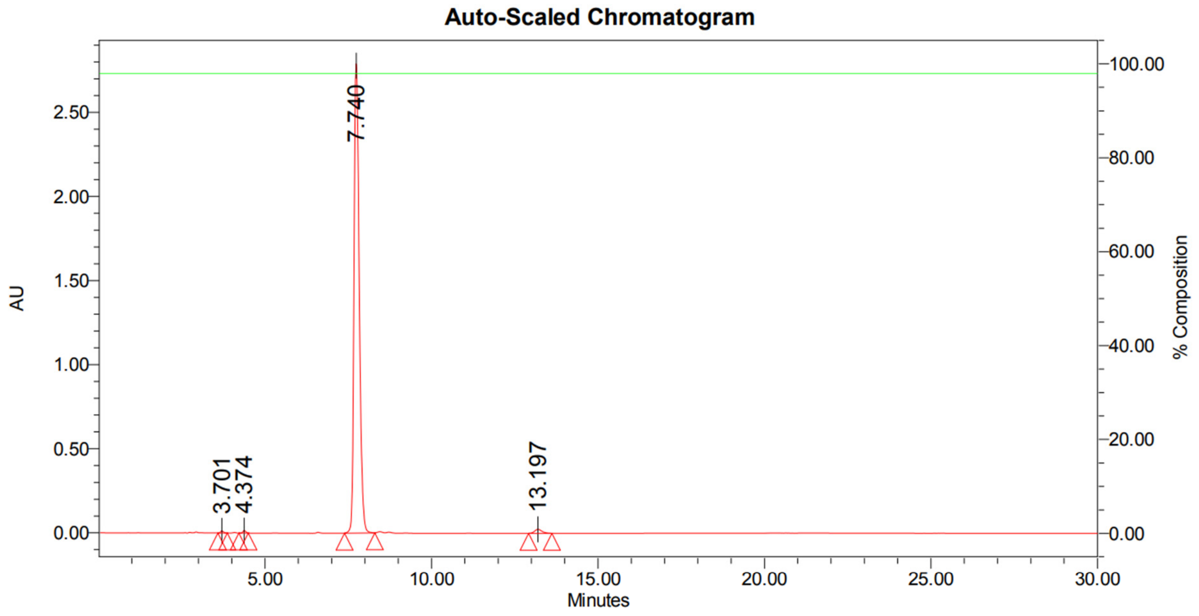

Processed Channel: PDA 254.0 nm

	Processed Channel	Retention Time (min)	Area	% Area	Height
1	PDA 254.0 nm	3.701	75189	0.24	12824
2	PDA 254.0 nm	4.374	91093	0.29	15355
3	PDA 254.0 nm	7.740	30364029	98.29	2801335
4	PDA 254.0 nm	13.197	362613	1.17	23742

^{1}H-NMR spectrum of 2-hydroxy-6-(pentadecyloxy)benzoic acid (1f).

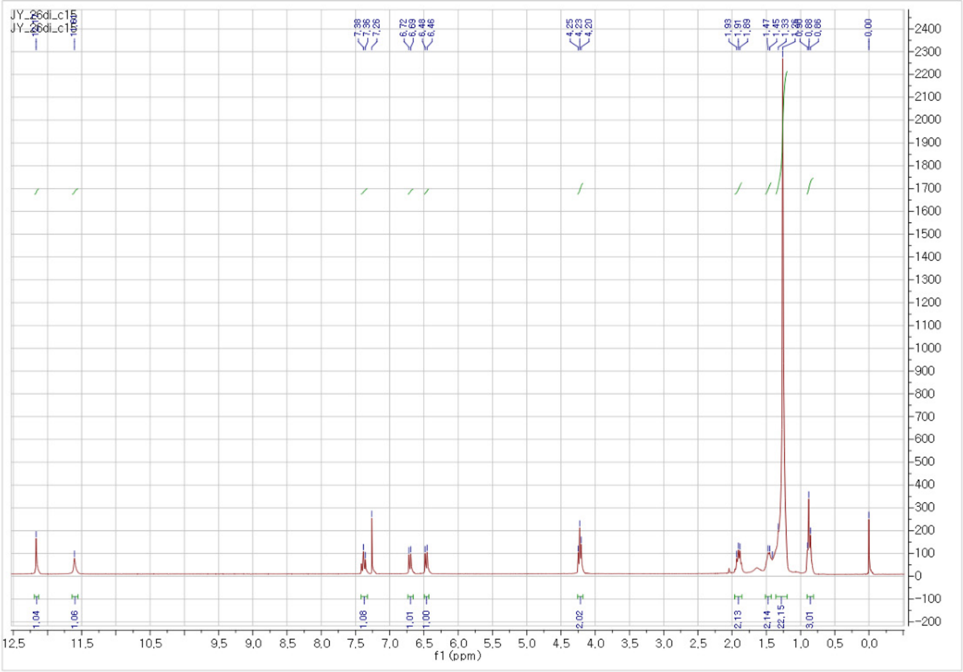

^{13}C-NMR spectrum of 2-hydroxy-6-(pentadecyloxy)benzoic acid (1f).

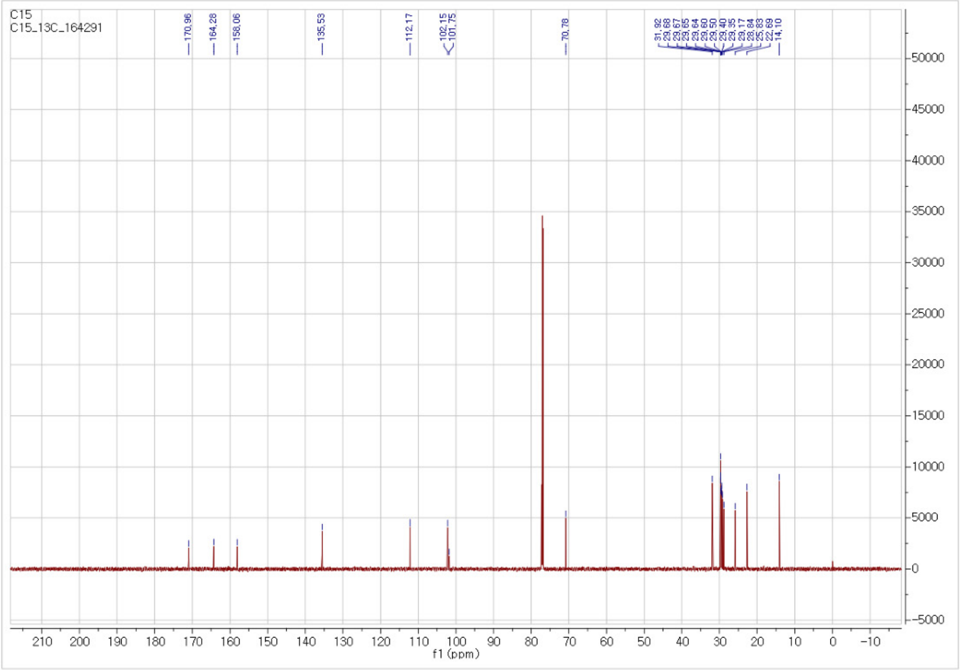

HRMS spectrum of 2-hydroxy-6-(pentadecyloxy)benzoic acid (1f).

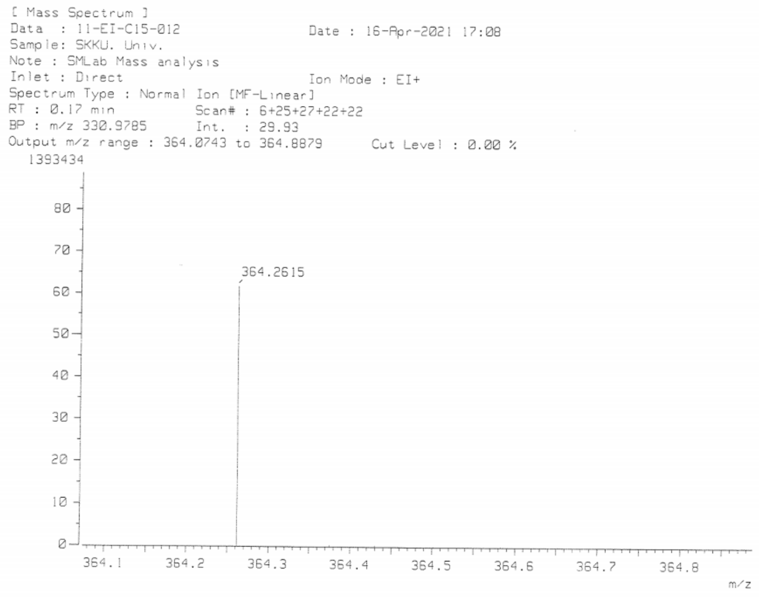

HPLC data of 2-hydroxy-6-(pentadecyloxy)benzoic acid (1f).

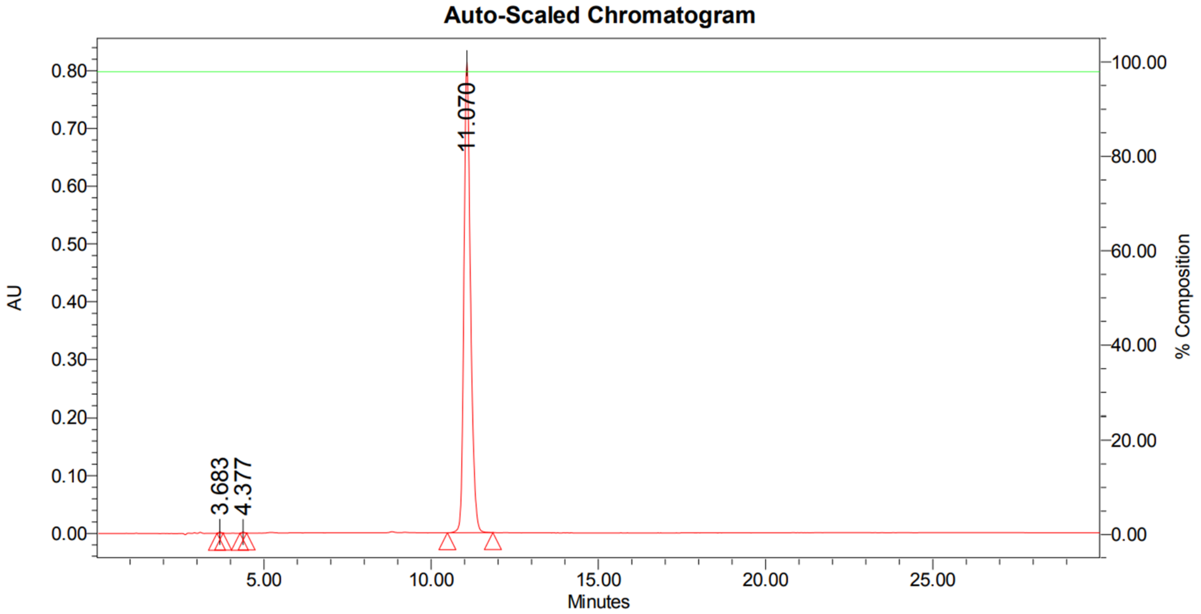

Processed Channel: PDA 254.0 nm

	Processed Channel	Retention Time (min)	Area	% Area	Height
1	PDA 254.0 nm	3.683	11423	0.10	2194
2	PDA 254.0 nm	4.377	11196	0.10	1838
3	PDA 254.0 nm	11.070	11107763	99.80	814090

^{1}H-NMR spectrum of 7-hydroxy-2,2-dimethyl-4H-benzo[d][1,3]dioxin-4-one (8).

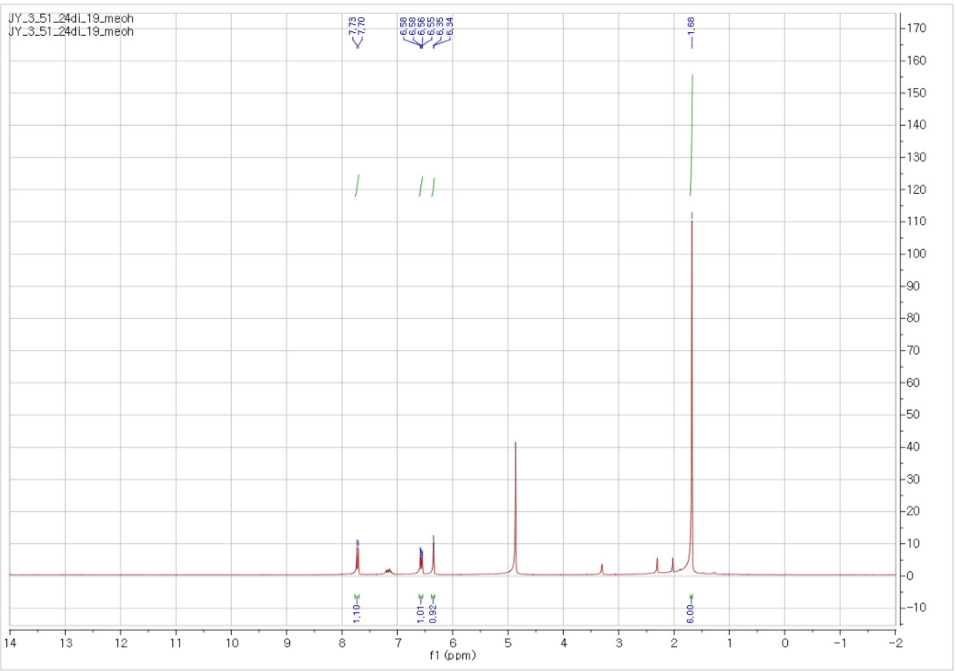

^{13}C-NMR spectrum of 7-hydroxy-2,2-dimethyl-4H-benzo[d][1,3]dioxin-4-one (8).

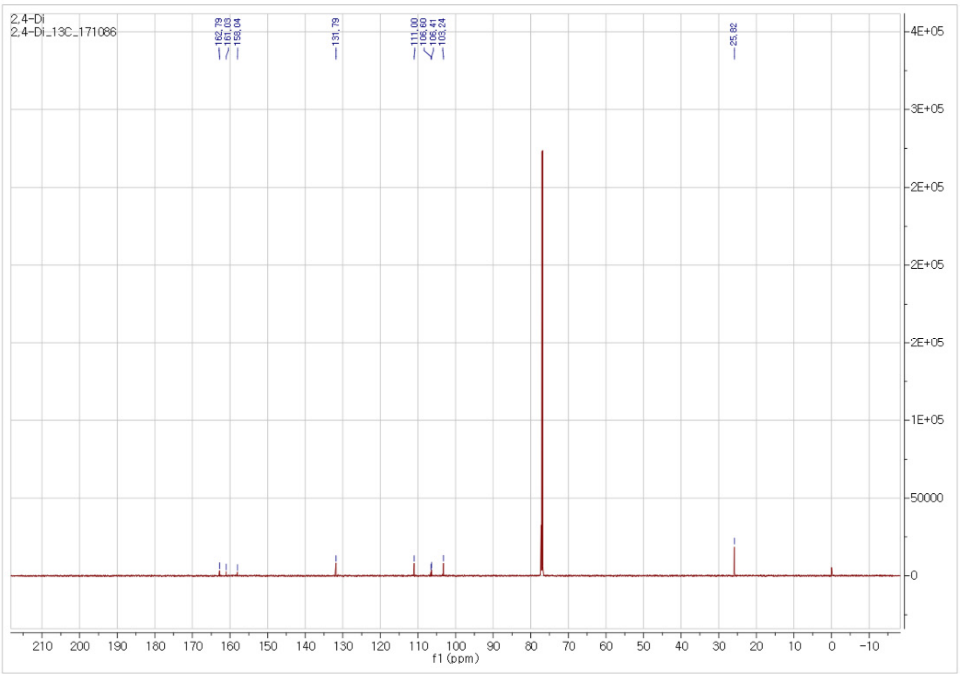

HRMS spectrum of 7-hydroxy-2,2-dimethyl-4H-benzo[d][1,3]dioxin-4-one (8).

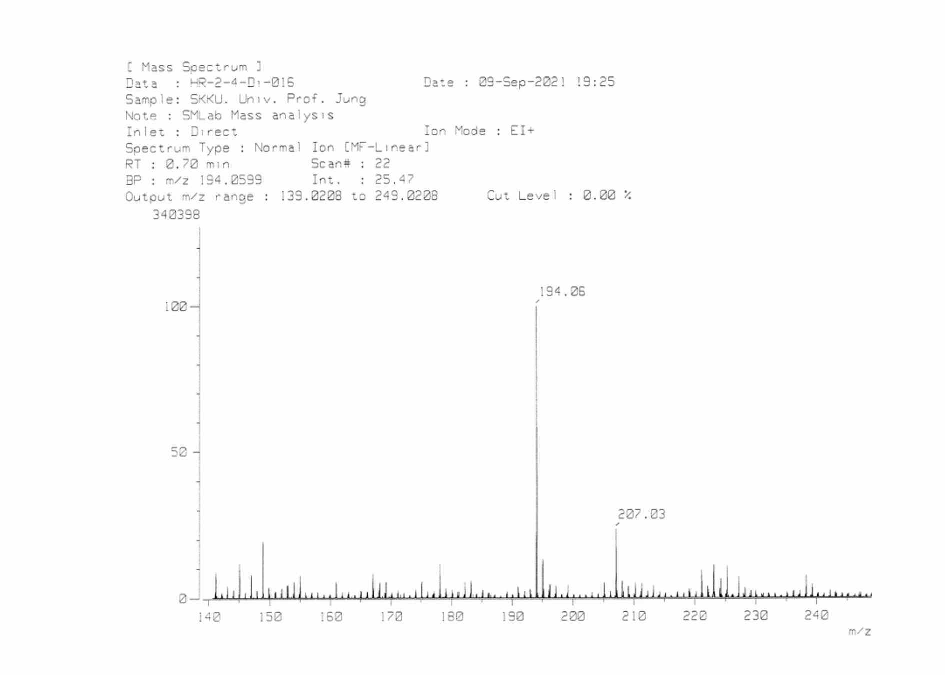

^{1}H-NMR spectrum of 2-hydroxy-4-(tridecyloxy)benzoic acid (2) .

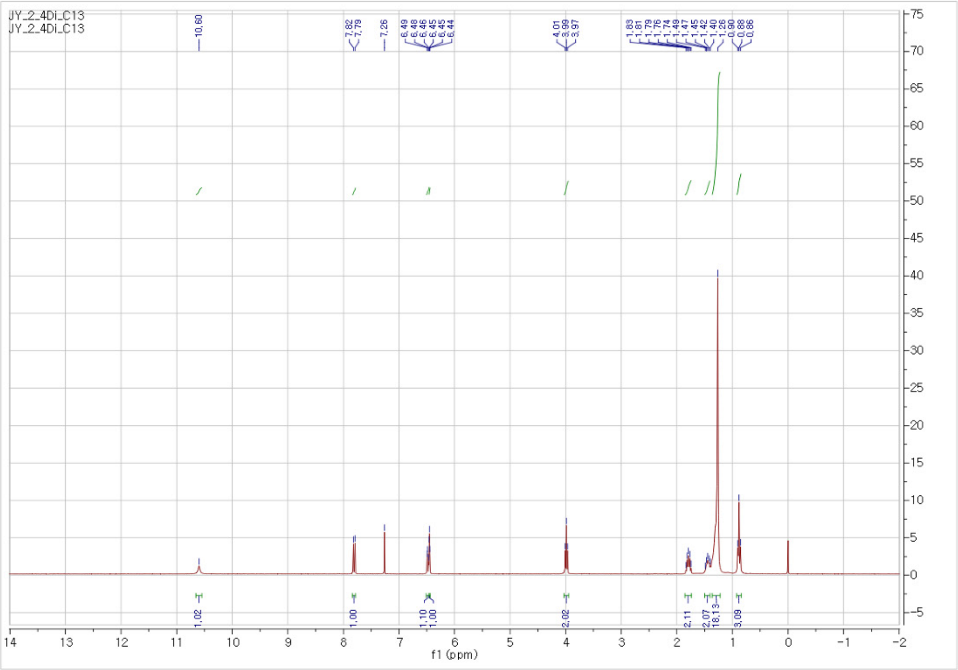

^{13}C-NMR spectrum of 2-hydroxy-4-(tridecyloxy)benzoic acid (2).

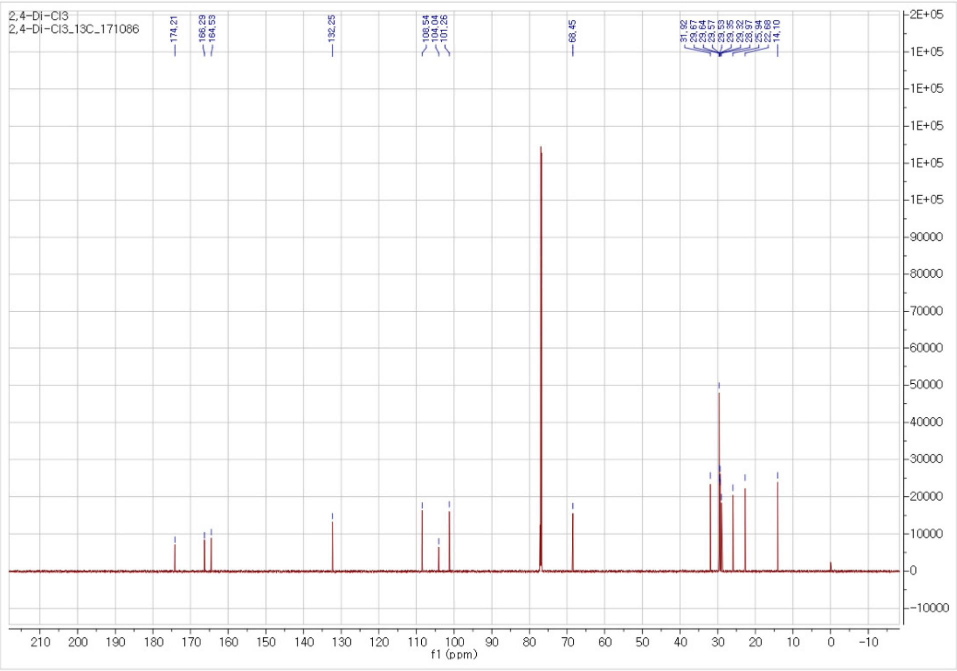

HRMS spectrum of 2-hydroxy-4-(tridecyloxy)benzoic acid (2).

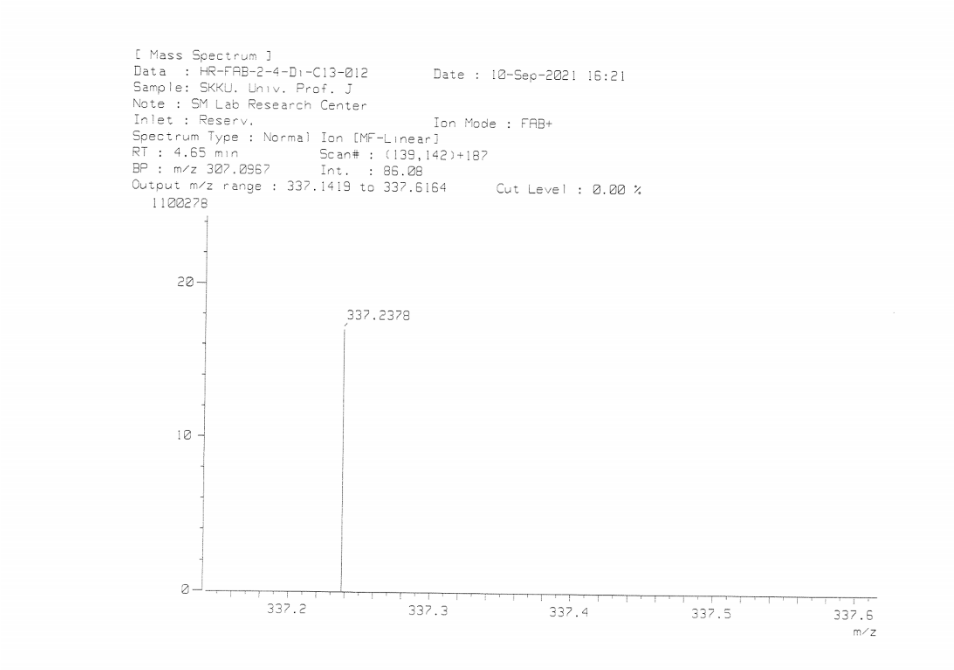

HPLC data of 2-hydroxy-4-(tridecyloxy)benzoic acid (2).

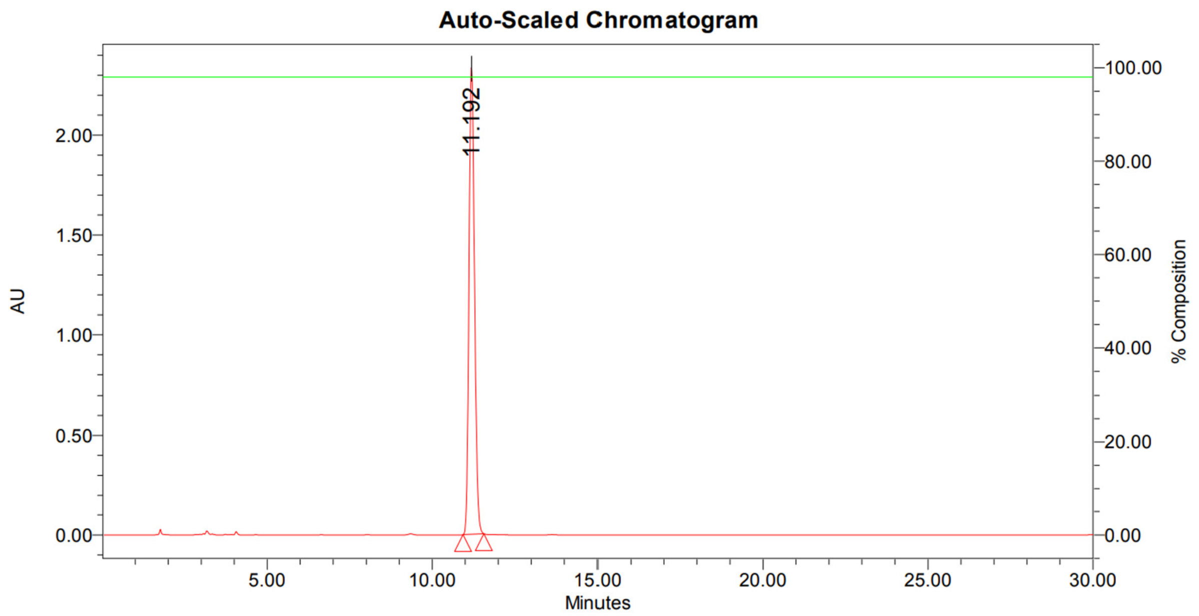

Processed Channel: PDA 254.0 nm

	Processed Channel	Retention Time (min)	Area	% Area	Height
1	PDA 254.0 nm	11.192	27346788	100.00	2346050

^{1}H-NMR spectrum of 6-hydroxy-2,2-dimethyl-4H-benzo[d][1,3]dioxin-4-one (11).

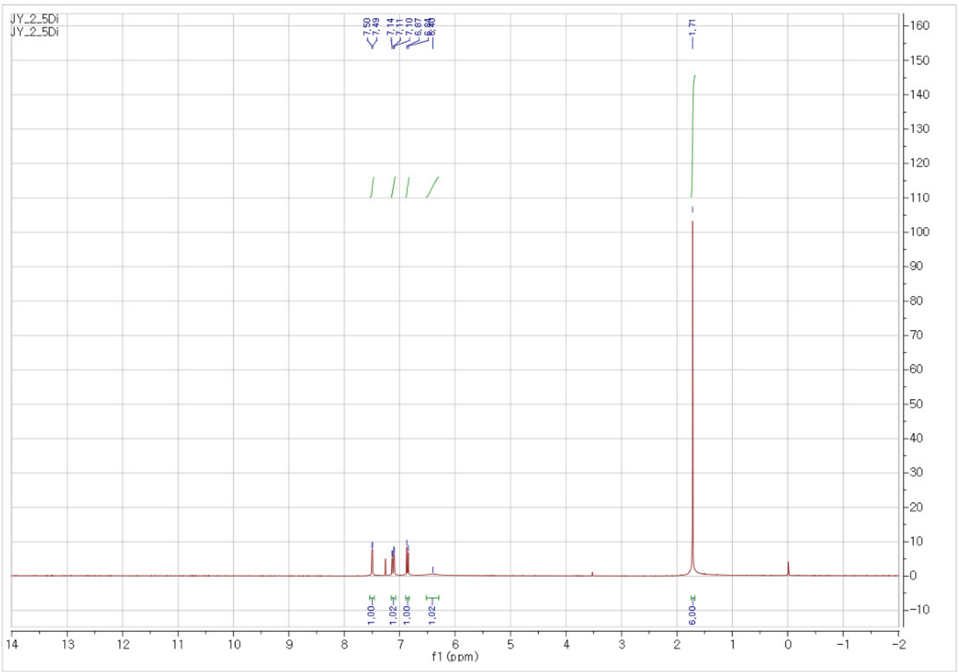

^{13}C-NMR spectrum of 6-hydroxy-2,2-dimethyl-4H-benzo[d][1,3]dioxin-4-one (11).

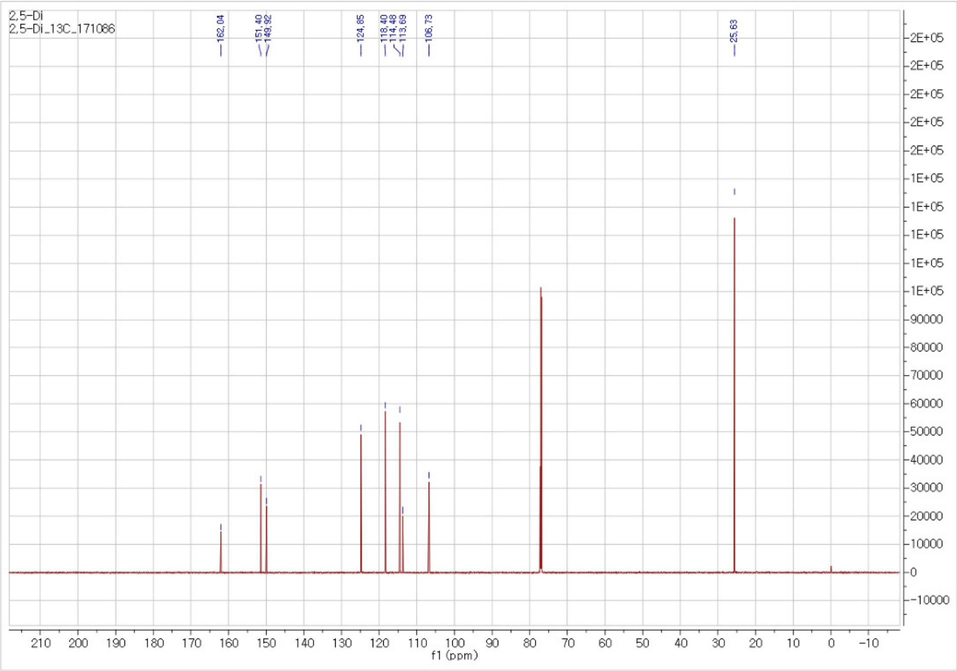

HRMS spectrum of 6-hydroxy-2,2-dimethyl-4H-benzo[d][1,3]dioxin-4-one (11).

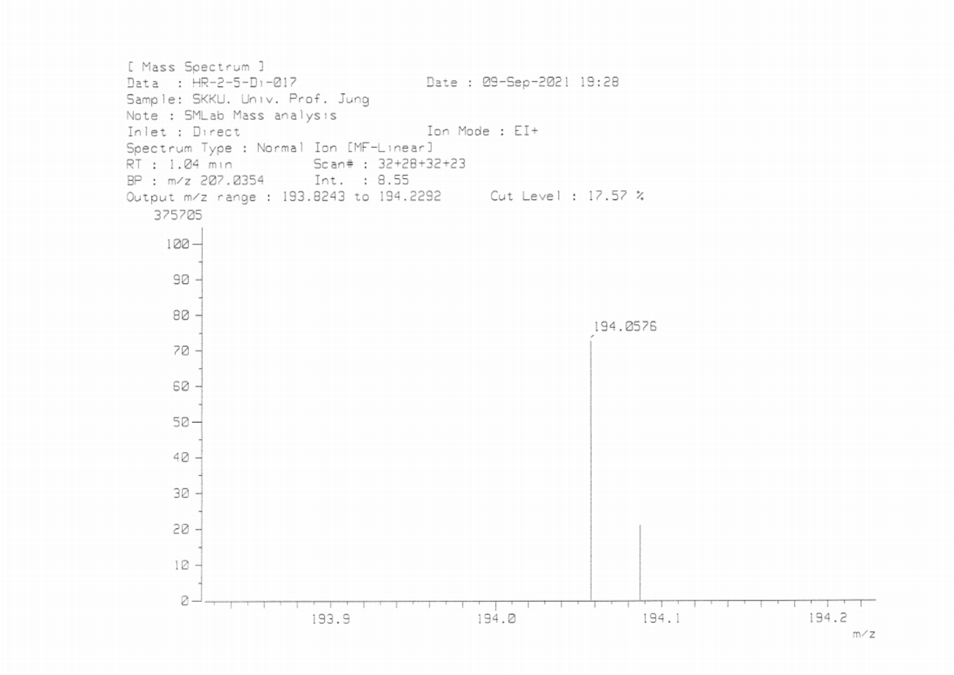
[Mass Spectrum]
Data : HR-2-5-Di-017
Date : 09-Sep-2021 19:28
Sample: SKKU. Univ. Prof. Jung
Note : SMLab Mass analysis
Inlet : Direct
Ion Mode : EI+
Spectrum Type : Normal Ion [MF-Linear]
RT : 1.04 min
Scan# : 32+28+32+23
BP : m/z 207.0354
Int. : 8.55
Output m/z range : 193.8243 to 194.2292
Cut Level : 17.57 %
375705
194.0576
m/z

^{1}H-NMR spectrum of 2-hydroxy-5-(tridecyloxy)benzoic acid (3) .

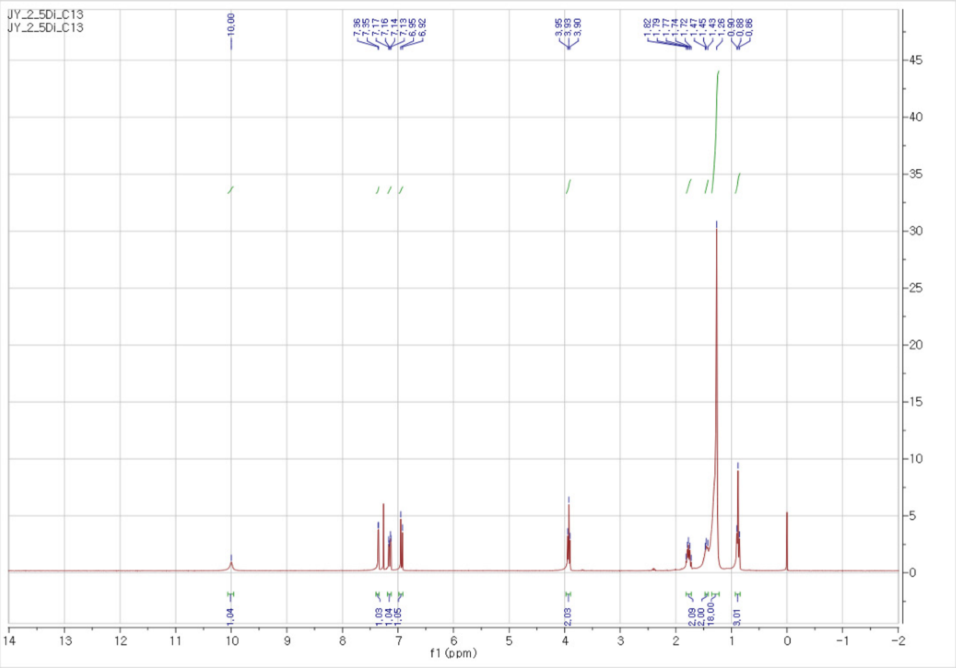

^{13}C-NMR spectrum of 2-hydroxy-5-(tridecyloxy)benzoic acid (3) .

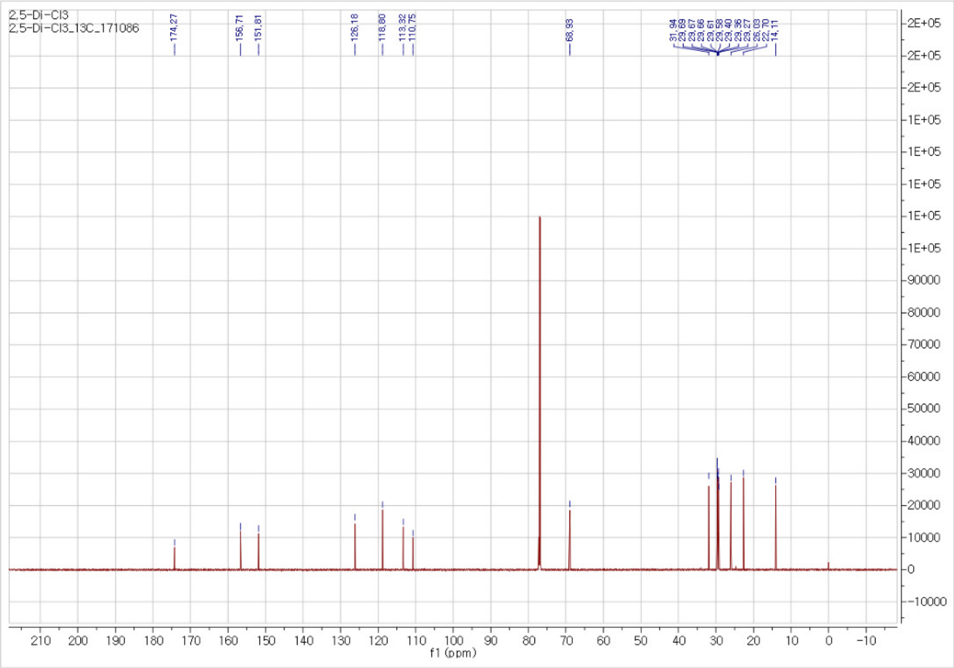

HRMS spectrum of 2-hydroxy-5-(tridecyloxy)benzoic acid (3).

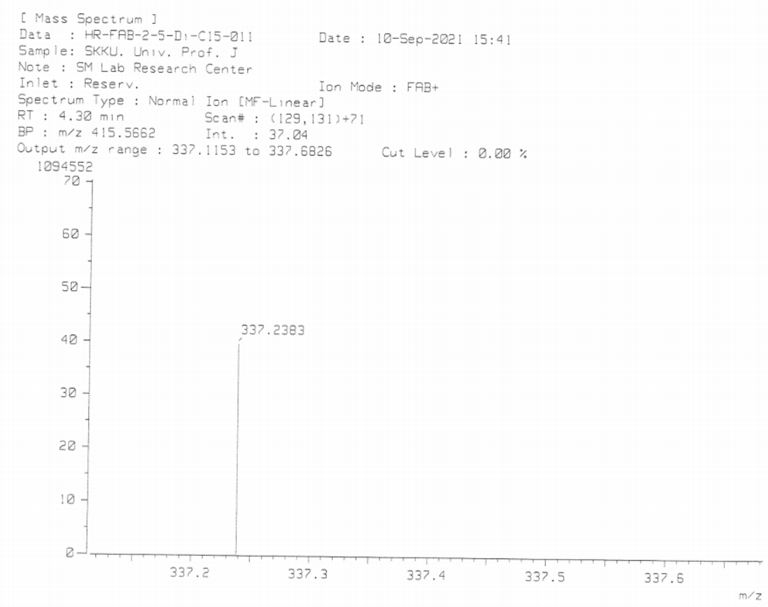

HPLC data of 2-hydroxy-5-(tridecyloxy)benzoic acid (3).

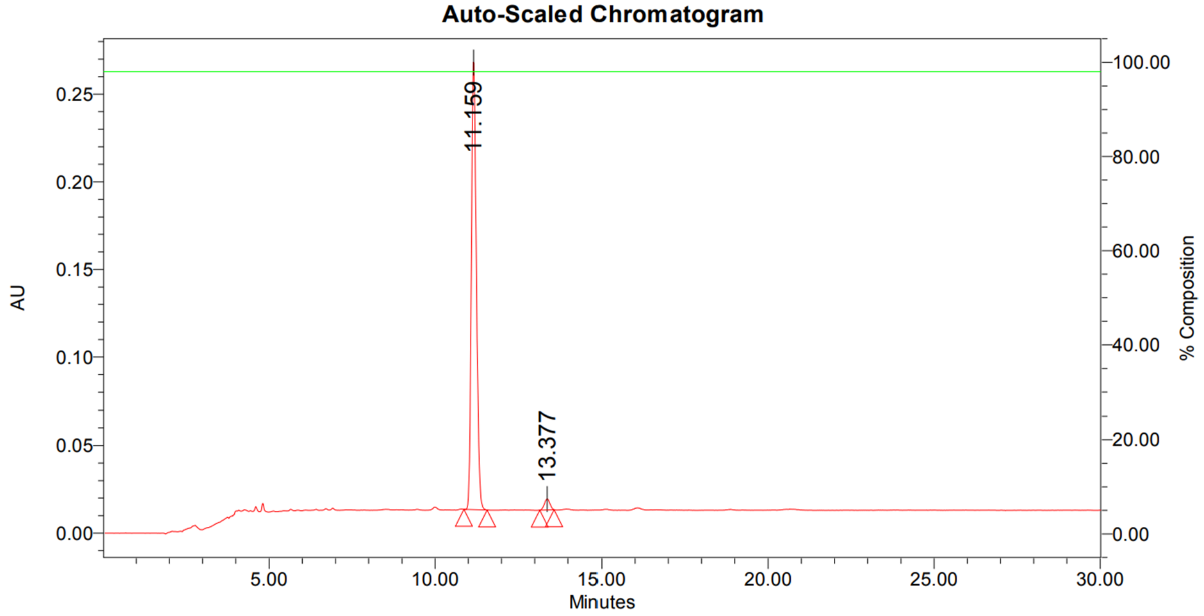

Processed Channel: PDA 254.0 nm

	Processed Channel	Retention Time (min)	Area	% Area	Height
1	PDA 254.0 nm	11.159	2683605	97.41	256634
2	PDA 254.0 nm	13.377	71331	2.59	6241
